# The Munich MIDY Pig Biobank – A unique resource for studying organ crosstalk in diabetes

**DOI:** 10.1016/j.molmet.2017.06.004

**Published:** 2017-06-13

**Authors:** Andreas Blutke, Simone Renner, Florian Flenkenthaler, Mattias Backman, Serena Haesner, Elisabeth Kemter, Erik Ländström, Christina Braun-Reichhart, Barbara Albl, Elisabeth Streckel, Birgit Rathkolb, Cornelia Prehn, Alessandra Palladini, Michal Grzybek, Stefan Krebs, Stefan Bauersachs, Andrea Bähr, Andreas Brühschwein, Cornelia A. Deeg, Erica De Monte, Michaela Dmochewitz, Caroline Eberle, Daniela Emrich, Robert Fux, Frauke Groth, Sophie Gumbert, Antonia Heitmann, Arne Hinrichs, Barbara Keßler, Mayuko Kurome, Miriam Leipig-Rudolph, Kaspar Matiasek, Hazal Öztürk, Christiane Otzdorff, Myriam Reichenbach, Horst Dieter Reichenbach, Alexandra Rieger, Birte Rieseberg, Marco Rosati, Manuel Nicolas Saucedo, Anna Schleicher, Marlon R. Schneider, Kilian Simmet, Judith Steinmetz, Nicole Übel, Patrizia Zehetmaier, Andreas Jung, Jerzy Adamski, Ünal Coskun, Martin Hrabě de Angelis, Christian Simmet, Mathias Ritzmann, Andrea Meyer-Lindenberg, Helmut Blum, Georg J. Arnold, Thomas Fröhlich, Rüdiger Wanke, Eckhard Wolf

**Affiliations:** 1 Institute of Veterinary Pathology at the Centre for Clinical Veterinary Medicine, LMU Munich, Veterinärstr. 13, D-80539 Munich, Germany; 2 Chair for Molecular Animal Breeding and Biotechnology, Gene Center and Department of Veterinary Sciences, and Center for Innovative Medical Models (CiMM), LMU Munich, Feodor-Lynen-Str. 25, D-81377 Munich, Germany; 3 German Center for Diabetes Research (DZD), Ingolstädter Landstr. 1, D-85764 Neuherberg, Germany; 4 Laboratory for Functional Genome Analysis (LAFUGA), Gene Center, LMU Munich, Feodor-Lynen-Str. 25, D-81377 Munich, Germany; 5 German Mouse Clinic (GMC), Institute of Experimental Genetics, Helmholtz Zentrum München, Ingolstädter Landstr. 1, D-85764 Neuherberg, Germany; 6 Genome Analysis Center (GAC), Institute of Experimental Genetics, Helmholtz Zentrum München, Ingolstädter Landstr. 1, D-85764 Neuherberg, Germany; 7 Paul Langerhans Institute Dresden of the Helmholtz Zentrum München at the University Hospital and Faculty of Medicine Carl Gustav Carus of TU Dresden, Fetscherstr. 74, D-01307 Dresden, Germany; 8 Animal Physiology, Institute of Agricultural Sciences, ETH Zurich, Universitätsstr. 2, CH-8092 Zurich, Switzerland; 9 Clinic for Small Animal Surgery and Reproduction, Center for Clinical Veterinary Medicine, LMU Munich, Veterinärstr. 13, D-80539 Munich, Germany; 10 Experimental Ophthalmology, Philipps University of Marburg, Baldingerstr., D-35033 Marburg, Germany; 11 Institute for Infectious Diseases and Zoonosis, LMU Munich, Veterinärstr. 13, D-80539 Munich, Germany; 12 Clinic for Swine at the Centre of Clinical Veterinary Medicine, LMU Munich, Sonnenstr. 16, D-85764 Oberschleißheim, Germany; 13 Munich Center of NeuroSciences – Brain & Mind, Großhaderner Str. 2, D-82152 Planegg-Martinsried, Germany; 14 Bavarian State Research Center for Agriculture – Institute for Animal Breeding, Prof.-Dürrwaechter-Platz 1, D-85586 Grub-Poing, Germany; 15 Chair for Animal Physiology, Department of Veterinary Sciences, LMU Munich, Veterinärstr. 13, D-80539 Munich, Germany; 16 Institute of Pathology, LMU Munich, Thalkirchner Str. 36, D-80337 Munich, Germany; 17 Chair of Experimental Genetics, School of Life Science Weihenstephan, Technische Universität München, Ingolstädter Landstr. 1, D-85764 Neuherberg, Germany; 18 MWM Biomodels GmbH, Hauptstr. 41, D-84184 Tiefenbach, Germany

**Keywords:** MIDY, Hyperglycemia, Insulin insufficiency, Pig model, Biobank, Random systematic sampling, Transcriptomics, Proteomics, Metabolomics, Stereology, CE, cholesterol ester, CPT1, carnitine O-palmitoyltransferase 1, ER, endoplasmic reticulum, FFA, free fatty acids, MIDY, mutant *INS* gene-induced diabetes of youth, PC, phosphatidylcholine, PCA, principal component analysis, SM, sphingomyelin, TAG, triacylglycerol, WT, wild-type

## Abstract

**Objective:**

The prevalence of diabetes mellitus and associated complications is steadily increasing. As a resource for studying systemic consequences of chronic insulin insufficiency and hyperglycemia, we established a comprehensive biobank of long-term diabetic *INS*^C94Y^ transgenic pigs, a model of mutant *INS* gene-induced diabetes of youth (MIDY), and of wild-type (WT) littermates.

**Methods:**

Female MIDY pigs (n = 4) were maintained with suboptimal insulin treatment for 2 years, together with female WT littermates (n = 5). Plasma insulin, C-peptide and glucagon levels were regularly determined using specific immunoassays. In addition, clinical chemical, targeted metabolomics, and lipidomics analyses were performed. At age 2 years, all pigs were euthanized, necropsied, and a broad spectrum of tissues was taken by systematic uniform random sampling procedures. Total beta cell volume was determined by stereological methods. A pilot proteome analysis of pancreas, liver, and kidney cortex was performed by label free proteomics.

**Results:**

MIDY pigs had elevated fasting plasma glucose and fructosamine concentrations, C-peptide levels that decreased with age and were undetectable at 2 years, and an 82% reduced total beta cell volume compared to WT. Plasma glucagon and beta hydroxybutyrate levels of MIDY pigs were chronically elevated, reflecting hallmarks of poorly controlled diabetes in humans. In total, ∼1900 samples of different body fluids (blood, serum, plasma, urine, cerebrospinal fluid, and synovial fluid) as well as ∼17,000 samples from ∼50 different tissues and organs were preserved to facilitate a plethora of morphological and molecular analyses. Principal component analyses of plasma targeted metabolomics and lipidomics data and of proteome profiles from pancreas, liver, and kidney cortex clearly separated MIDY and WT samples.

**Conclusions:**

The broad spectrum of well-defined biosamples in the Munich MIDY Pig Biobank that will be available to the scientific community provides a unique resource for systematic studies of organ crosstalk in diabetes in a multi-organ, multi-omics dimension.

## Highlights

• MIDY pigs represent a model of poorly controlled diabetes mellitus (DM) in humans.
• A complex biobank was built from 2-year-old MIDY and wild-type pigs using the principles of random systematic sampling.
• Targeted metabolomics and lipidomics analyses of plasma samples revealed clear separation of MIDY and wild-type pigs.
• The Munich MIDY Pig Biobank facilitates systematic studies of organ crosstalk in DM in a multi-organ, multi-omics dimension.

## 1 Introduction

Diabetes mellitus is a complex metabolic disease with markedly increasing prevalence worldwide (http://www.diabetes.org/diabetes-basics/statistics/). Acute hyperglycemia may lead to life-threatening diabetic ketoacidosis, chronic hyperglycemia is associated with macrovascular complications, increasing the risk for myocardial infarction and stroke, and microvascular complications leading to diabetic nephropathy, retinopathy, and neuropathy (reviewed in Ref. [1]). The molecular disease mechanisms behind these multi-organ changes are only partially understood.

Molecular profiling techniques on the transcriptome, proteome, and metabolome levels facilitate the investigation of intermediate molecular phenotypes in disease-related cells, tissues, and organs (reviewed in Ref. [2]). Systems biology approaches such as integrative analyses of multi-omics data sets aim to provide novel mechanistic insights and to identify therapeutic targets and biomarkers.

Central gene expression data repositories such as NCBI Gene Expression Omnibus (GEO, http://www.ncbi.nlm.nih.gov/geo/) and EMBL-EBI ArrayExpress Archive (http://www.ebi.ac.uk/arrayexpress/) are important sources for capturing transcriptome alterations in diabetic patients (e.g. Ref. [3]), but are mostly limited to one or few tissues per study (e.g. blood cells and adipose tissue in Ref. [4]). Recently, the Human Diabetes Proteome Project (HDPP) was launched with an initial focus on islets of Langerhans, insulin-producing cell lines, and blood samples from diabetes-related patient cohorts [5]. Moreover, targeted and non-targeted metabolomics approaches are available for diabetes research and have been used for analyzing human samples and samples from model organisms (reviewed in Ref. [6]).

Although cross-tissue networks with a limited spectrum of tissues have been constructed in several studies, integration of multi-omics data with expanded tissue coverage would markedly benefit disease-related network analyses on an organism-wide scale [2]. This is particularly true for metabolic diseases such as diabetes and obesity, for which multiple tissues/organs may be causally involved in and/or affected by disease-relevant tissue crosstalk (reviewed in Ref. [7]).

For ethical reasons, the spectrum of tissues available from diabetic patients is limited. In addition, confounding factors such as age, comorbidities, and variance introduced by tissue sampling and storage procedures may complicate the analysis and interpretation of omics data from human samples. Samples from diabetic rodent models are less variable, but the amount of tissue available for multi-omics analyses is limited.

Pigs are interesting models for diabetes and obesity research and can be genetically engineered to mimic human disease mechanisms (reviewed in Ref. [8]). Transgenic pigs expressing the mutant insulin C94Y are a model for permanent neonatal diabetes [9], now termed mutant *INS* gene-induced diabetes of youth (MIDY) (reviewed in Ref. [10]). Corresponding *INS*/*Ins2* mutations that disrupt the C(B7)-C(A7) interchain disulfide bond of the insulin molecule exist also in humans and in the widely used Akita mouse model (reviewed in Ref. [10]). Expression of the mutant *INS*/*Ins2* leads to impaired trafficking of normal proinsulin by formation of high-molecular weight complexes with misfolded (pro)insulin, accumulation of misfolded insulin in the endoplasmic reticulum (ER), and ER stress, which finally triggers beta-cell apoptosis (reviewed in Ref. [10]). Accordingly, MIDY pigs are characterized by impaired insulin secretion, increased fasting glucose levels, and progressively decreasing beta cell mass [9].

To generate a unique resource for studying consequences of chronic insulin insufficiency and hyperglycemia in a multi-tissue, multi-omics approach, we generated a complex biobank of more than 50 different tissues and body fluids from two-year-old MIDY pigs and WT littermate controls (highlighted in Ref. [11]). A comprehensive standardized protocol, taking the principles of systematic uniform random sampling into account, was established [12] to ensure uniform high quality of representative samples for a broad spectrum of analyses, including molecular profiling as well as qualitative and quantitative morphological investigations.

## 2 Material and methods

### 2.1 MIDY pig model

A cohort of 4 female MIDY pigs and 5 female WT littermates was maintained for two years. Animals were housed under controlled conditions and had a once-daily feeding regimen (Supplementary Figure 1a) and free access to water. Treatment of MIDY pigs with a combination of long-acting insulin (Lantus^®^; Sanofi) and short-acting insulin (NovoRapid^®^; NovoNordisk) was started at age 2 months aiming for moderate hyperglycemic levels to mimic suboptimal insulin treatment (Supplementary Figure 1b). Blood glucose levels were determined once or twice daily using a Precision Xceed^®^ glucometer and Precision XtraPlus^®^ test stripes (Abbott) to control treatment [9] (Supplementary Figure 1c). WT and MIDY sows were estrus synchronized [13] and inseminated 12 days prior to necropsy to exclude estrous cycle related effects on molecular profiles of tissues and body fluids and to facilitate collection of conceptuses. All experiments were performed according to the German Animal Welfare Act with permission from the responsible authority (Government of Upper Bavaria), following the ARRIVE guidelines and Directive 2010/63/EU for animal experiments.

### 2.2 Metabolic characterization, clinical chemistry, targeted metabolomics, and lipidomics

Blood samples were taken regularly using EDTA coated tubes (Monovette^®^ blood collection system, Sarstedt). Plasma was separated by centrifugation and stored at −80 °C. Plasma insulin, C-peptide and glucagon levels were determined using specific RIAs (Merck Millipore) or ELISAs (Mercodia). Clinical chemical parameters in plasma were determined using an AU400 (Olympus) or AU480 autoanalyzer (Beckman–Coulter) and adapted reagent kits from Olympus, Beckman–Coulter, or Sentinel (fructosamine).

Targeted metabolomics analysis of plasma samples was done by liquid chromatography-electrospray ionization-tandem mass spectrometry and flow injection analysis-electrospray ionization tandem mass spectrometry measurements using the Absolute*IDQ*^TM^ p180 Kit (Biocrates Life Sciences AG, Innsbruck, Austria). Out of 10 μL plasma 188 metabolites were quantified (for details, see Ref. [14] and Supplementary Table 2). Concentrations of all metabolites were calculated using internal standards and are reported in μM. Lipid extraction and shotgun mass spectrometry analysis was performed by Lipotype GmbH as described [15].

### 2.3 Establishment of the Munich MIDY Pig Biobank

Two-year-old MIDY (n = 4) and WT pigs (n = 5) were clinically examined the day before necropsy. Thereby general condition, nutritional status, body posture, body temperature, skin, hair coat, mucus membranes, conjunctiva, and feces were evaluated. Additionally, auscultation was performed to determine heart and breathing frequency and exclude pathological heart and respiratory noises. Overnight fasted pigs were anesthetized by intramuscular injection of ketamine (Ursotamin^®^, Serumwerk Bernburg) and azaperone (Stresnil^®^, Elanco Animal Health) followed by intravenous application of ketamine and xylazine (Xylazin 2%, Serumwerk Bernburg). Blood samples were taken by cardio puncture. Animals were then euthanized under anesthesia by intravenous injection of T61^®^ (Intervet) and immediately subjected to necropsy. Body weight and length (tip of nose to base of tail) as well as weights and dimensions of internal organs were determined. To ensure generation of representative, high-quality tissue samples, suitable for a broad range of analyses, standardized sampling procedures [12] were used. From complex organs with several morphologically and/or functionally distinct compartments, such as brain or heart, reproducible samples were taken from standardized, deliberately chosen, anatomic locations in defined orientations. For parenchymatous organs, such as lungs, liver, spleen, kidney, or pancreas, systematic random sampling regimes [12] were employed. Samples taken from the selected locations were fractionated and differentially processed according to the demands of various analytical methods (Table 1). Samples designated for molecular profiling analyses were collected and shock frozen to −80 °C within a period of maximal 20 min after death of the animal.

**Table 1:** Overview of samples collected in the Munich MIDY Pig Biobank.

Organ system	Organ/tissue	Number samples per organ/tissue compartment	Samples	Downstream analyses
Cardiovascular system	Heart	Right and left ventricular (38) and atrial (20) myocardium, heart valves (8)	PE, EL, CRYO, −80 °C	
	Blood vessels	Thoracic and abdominal aorta (24), carotid arteries (12), jugular veins (12), coronary vessels (20)	PE, EL, CRYO, −80 °C	
Respiratory tract (RT)	Upper RT	Nasal septum (2), larynx (1), trachea (á 2 samples of the proximal, medial, and distal part)	PE	
	Lung	Lung parenchyma (90), main bronchi (8)	PE, EL, CRYO, −80 °C	
Hepato-pancreatic system	Liver	Liver parenchyma (74), gall bladder (1)	PE, EL, CRYO, −80 °C	
	Pancreas	Pancreas parenchyma (100), pancreaticoduodenal lymph node (2)	PE, EL, CRYO, −80 °C	
Gastro-intestinal tract	Tongue, salivary glands, esophagus	Tongue (4), mandibular gland (2), parotid gland (2), esophagus (á 4 samples of the proximal and distal part)	PE	
	Stomach	Cardiac portion, fundus, and pyloric portion (á 56 samples)	PE, PlE, EL, CRYO, −80 °C	
	Intestine	Jejunum (70), duodenum, ileum, cecum, colon (á 35 samples), ileal papilla (1), mesenteric (2) and Ileocolic (2) lymph nodes	PE, PlE, EL, CRYO, −80 °C	
	Ingesta/feces	Stomach, duodenum, jejunum, ileum, cecum, colon (á 10 samples).	−80 °C	
Uro-genital system	Kidney	Fresh- and perfusion-fixed tissue: cortex (55), outer- (50), and inner zone of the renal medulla (50)	PE, PlE, EL, CRYO, −80 °C	
	Lower urinary tract	Ureter (á 2 samples of the proximal, medial, and distal part), urinary bladder (corpus: 4, trigone: 2 samples), urethra (2)	PE	
	Genital tract	Ovary (6), uterus (24), vagina (9)	PE, EL, CRYO	
Immune and hematopoietic system	Spleen, thymus, bone marrow, tonsil, peripheral lymph nodes	Spleen (24), thymus (2), sternal bone marrow (2), tonsil (2), superficial inguinal lymph nodes (7), axillary lymph nodes (2)	PE, CRYO, −80 °C	
Endocrine system	Thyroid gland, pituitary gland, adrenal gland	Thyroid gland (20), pituitary gland (2), adrenal gland (1)	PE, CRYO, −80 °C	
Nervous system	Brain	Neocortex (2), cerebellar cortex (2), caudate nucleus (2), thalamus (2), hippocampus (2), hypothalamus (2), pons (2), frontal brain standard histology sections (6), trigeminal ganglia (2)	PE, −80 °C	
	Nerves	Vagus nerve, sciatic nerve, common fibular nerve, radial nerve (proximal and distal part), ulnar nerve (proximal and distal part), tibial nerve (á 11 samples), sympathetic trunk (5)	PE, EL, −80 °C	
	Spinal cord	Cervical spinal cord, thoracic intumescence region, lumbar intumescence region (á 12 samples), dorsal root ganglia (DRG, á 9 samples of thoracic and lumbar DRG)	PE, CRYO, −80 °C	
Integument	Skin	Inner thigh (18), perineum (9), snout (9), hoofs (á 1 sample of the medial and lateral hoofs of the front and hind legs)	PE, PlE, EL, CRYO, −80 °C	
	Mammary gland	Cranial and penultimate complex (á 8 samples)	PE, −80 °C	
Adipose tissue	Subcutaneous and visceral adipose tissue	Subcutaneous adipose tissue (á 18 samples of the abdomen and back), visceral adipose tissue (á 18 samples of the mesenteric and perirenal adipose tissue)	PE, CRYO, −80 °C	
Musculo-skeletal system	Skeletal muscles	Triceps brachii muscle, (gluteo)biceps muscle, longissimus lumborum muscle, tibialis cranialis muscle, diaphragm (á 17 samples)	PE, PlE, EL, CRYO, −80 °C	
	Bones and joints	Femoral bone (1), radial bone (1), ulna (1, olecranon), tibial bone (1), synovial membrane of the knee joint (1)	PE	
Special senses	Eyes	Vitreous body (1), frontal portion of the globe (3), lens (3), retina (3), ocular fundus (2).	PE, −80 °C	
Body fluids		Urine (60), blood serum (60), blood plasma (60), cerebrospinal fluid (10), synovial fluid (4)	PE, PlE, EL, CRYO, −80 °C	

The indicated numbers of samples refer to the total numbers of individually collected specimen. As appropriate, samples were either taken from deliberately determined locations, or sampling locations were determined by systematic random sampling. **Sample processing**: Samples for morphologic analyses were fixed, using either 4% formaldehyde-solution, or 2.5%–6.25% glutaraldehyde solution, or Methacarn solution, or 96% ethanol. **PE**: Paraffin-embedding; **PlE**: Plastic embedding in GMA/MMA (glycolmethacrylate/methylmethacrylate); **EL**: Embedding in Epon-resin (glycid-ether) for preparation of semi-thin sections for (quantitative) morphological analyses and ultrathin sections for electron microscopy; **CRYO**: Preparation of frozen samples for cryo-histology; −**80 °C**: Cryopreservation of samples for molecular analyses. A detailed list of all individual Munich MIDY-Pig Biobank samples and the numbers of sampled locations per organ/tissue compartment is provided in Supplementary Table 3.

**Downstream analysis pictograms**: : Microscopy; : Electron microscopy; : (Quantitative) morphological analyses; : Molecular analyses (e.g. RNA-, protein-, metabolite profiling).

### 2.4 Quantification of beta cell volume

Pancreas samples were chosen by systematic random sampling and routinely processed for paraffin histology [16]. Beta cells were visualized using polyclonal guinea pig anti-porcine insulin antibodies (1:500; Dako), peroxidase-labeled rabbit anti-guinea pig antibodies (1:50; Dako), and diaminobenzidine as chromogen. Volume density and the total volume of beta cells within the pancreas were determined as described previously [16]. Multicolor immunofluorescence analysis was performed using mouse monoclonal anti-human insulin (1:3000, I2018, Sigma–Aldrich) and rabbit polyclonal anti-porcine glucagon (1:2000, BML-GA1181, Enzo) antibodies. All secondary antibodies were produced in donkey and coupled to AlexaFluor488 or Cy3 (Dianova). Embedding of slides was done with Vectashield antifade solution (Vector Laboratories) containing DAPI as a nuclear counterstain. Fluorescence analyses were performed using a confocal laser scanning microscope (LSM 710, Zeiss).

### 2.5 RNA extraction and RNA quality indices

RNA was extracted using TRIzol™ according to the manufacturer's instructions. In brief, frozen samples (80–150 mg) were crushed immediately after addition of 2 ml of pre-cooled TRIzol™ (Thermo Fisher Scientific) with a homogenizer (Silent Crusher M, Heidolph). Unlysed debris was removed by centrifugation, and 1 ml of the clear supernatant was mixed with 0.2 ml chloroform to induce phase separation. The aqueous phase was carefully recovered and cleared by centrifugation. RNA was precipitated by addition of 0.5 volumes of isopropanol to the aqueous phase and sedimented by centrifugation. Residual TRIzol™ was removed by two consecutive washing steps with 1 ml of 75% ethanol each. RNA was air dried for 5 min at room temperature and solved in DNase/RNase free water to a final concentration of 100–200 ng/μl. Each RNA solution was quantified by UV/VIS spectrometry (Nanodrop ND1000), and the 260/280 ratio was determined. The subsequent analysis of RNA integrity was performed on an Agilent Bioanalyzer 2100 (Agilent). If the automatic assignment of the RNA Integrity Number (RIN) failed, RIN was estimated by visual comparison of the RNA profile with those obtained from various RNA qualities.

### 2.6 Protein extraction and label free proteomics

Frozen tissue samples from pancreas, liver, and kidney cortex were homogenized as described previously [17]. Pierce 660 nm assay (Thermo Scientific) was used for total protein quantitation. From each sample, 100 μg of protein was reduced in 4 mM dithiothreitol (DTT) for 30 min at 56 °C and cysteine residues were blocked with 8 mM iodoacetamide (IAA) during a 30 min incubation in the dark. DTT to a final concentration of 10 mM was added to quench residual IAA during another 15 min incubation in the dark. Proteins were digested in two consecutive steps with 2 μg Lys-C (Wako) for 4 h at 37 °C and after dilution with water to a concentration of 1 M urea, with 2 μg porcine trypsin (Promega) overnight at 37 °C. LC-MS/MS analyses were performed on a TripleTOF 5600+ mass spectrometer (Sciex) as described [18]. Briefly, 1 μg of peptides were separated at 200 nl/min in consecutive linear gradients from 1 to 25% solvent B (0.1% formic acid in acetonitrile) in 120 min and from 25 to 50% solvent B in 10 min. Mass spectra were acquired using a data-dependent top 70 CID method. MS data processing was performed as published [18]. For database search, the sus scrofa subset of the UniProt database extended by the MaxQuant common contaminants database was used.

## 3 Results

### 3.1 MIDY pigs with limited insulin treatment represent a model of poorly controlled diabetes mellitus

**Figure 1:**
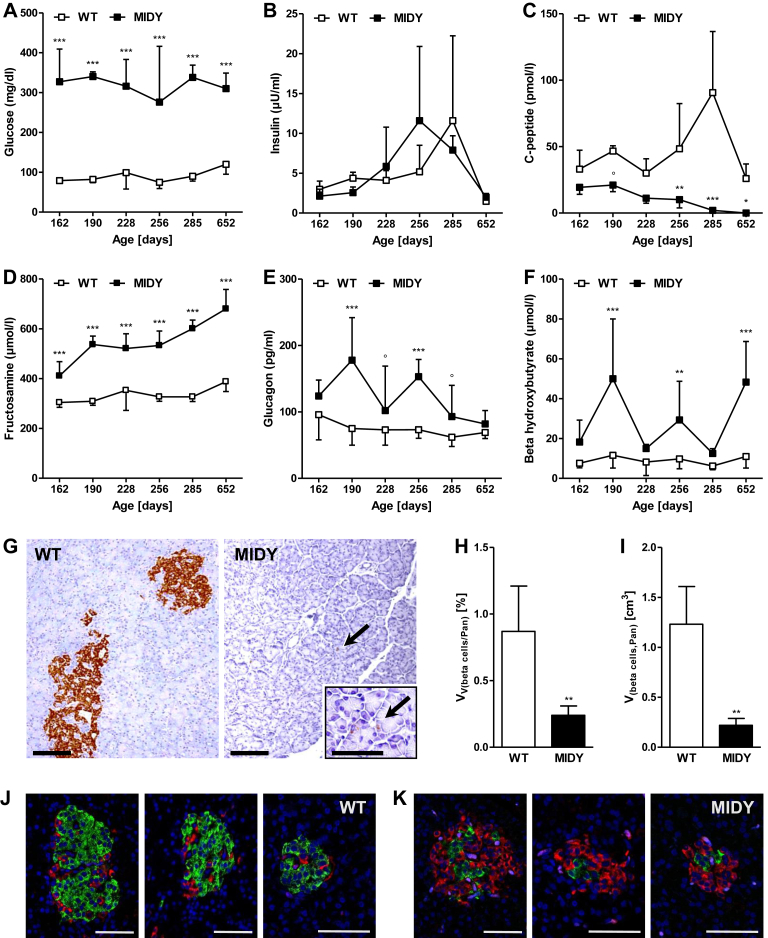
Metabolic characterization and beta cell volume of MIDY and WT pigs. **(A**–**F)** Age-related differences in fasting plasma concentrations of glucose **(A)**, insulin **(B)**, C-peptide **(C)**, fructosamine **(D)**, glucagon **(E)**, and beta hydroxybutyrate **(F)**. Fasting times were 18–24 h. Means and standard deviations are shown. Data were statistically evaluated by analysis of variance (Proc GLM, SAS 8.2), taking the effects of Group (MIDY, WT), Animal within Group, Age, and the interaction Group*Age into account. Significant differences between MIDY and WT pigs of the same age are indicated by asterisks (*p < 0.05; **p < 0.01; ***p < 0.001). Borderline significance (p < 0.08) is indicated by ° **(G**–**H)** Quantification of beta cell volume in MIDY and WT pancreas. Pancreas samples were chosen by systematic random sampling and routinely processed for paraffin histology. Volume density and the total volume of beta cells within the pancreas were determined as described in Material and methods. Detection of insulin by immunohistochemistry revealed drastically reduced areas of insulin-positive beta cell profiles in pancreas sections of MIDY pigs, as compared to WT pigs (**G**). Paraffin sections, chromogen: 3,3′-diaminobenzidine. Bars = 100 μm (and = 50 μm in inset). In MIDY pigs, the volume density (**H**), as well as the absolute volume of beta cells in the pancreas (**I**) is significantly smaller as in WT animals. Means and standard deviations are shown. Data were statistically evaluated by Student's t-tests. Significant differences between MIDY and WT pigs are indicated by asterisks (**p < 0.01). **(J**–**K)** Representative islets from WT **(J)** and MIDY pigs **(K)**. Insulin positive beta cells are stained with AlexaFluor488 (green), glucagon positive alpha cells are stained with Cy3 (red). Nuclei are stained with DAPI (blue). Bars = 50 μm.

Fasting plasma glucose levels of MIDY pigs were distinctly elevated (Figure 1A), although insulin treatment resulted in similar fasting insulin levels as in WT pigs (Figure 1B). Plasma C-peptide levels of MIDY pigs decreased with age and were undetectable at 2 years (Figure 1C). Plasma fructosamine levels were markedly elevated in MIDY pigs and increased with age (Figure 1D). Plasma glucagon levels were also increased in MIDY pigs (Figure 1E), as were beta hydroxybutyrate concentrations (Figure 1F).

Histological and stereological analyses of pancreas showed a significantly reduced volume density of beta cells in the pancreas (−72%; p < 0.01) and a significantly reduced total beta cell volume (−82%; p < 0.01) in MIDY compared to WT pigs (Figure 1G–I). In WT islets, beta cells are the most prevalent cell type. As in human islets [19], alpha cells are not only located in the periphery but also distributed inside the islets (Figure 1J). In MIDY islet profiles the proportion of alpha cells is markedly increased (Figure 1K).

Clinical-chemical analysis of terminal plasma samples revealed increased levels of total bilirubin (1.73 ± 0.44 μmol/l vs. 0.86 ± 0.21 μmol/l; p = 0.0224), increased alkaline phosphatase activity (106.00 ± 27.78 U/l vs. 36.00 ± 7.62 U/l; p = 0.0124), and reduced concentrations of creatinine (148 ± 14 μmol/l vs. 177 ± 14 μmol/l; p = 0.0151) and chloride (97.33 ± 1.41 mmol/l vs. 100.70 ± 1.49 mmol/l; p < 0.0109) in MIDY compared to WT pigs. The full set of clinical-chemical data is shown in Supplementary Table 1.

**Figure 2:**
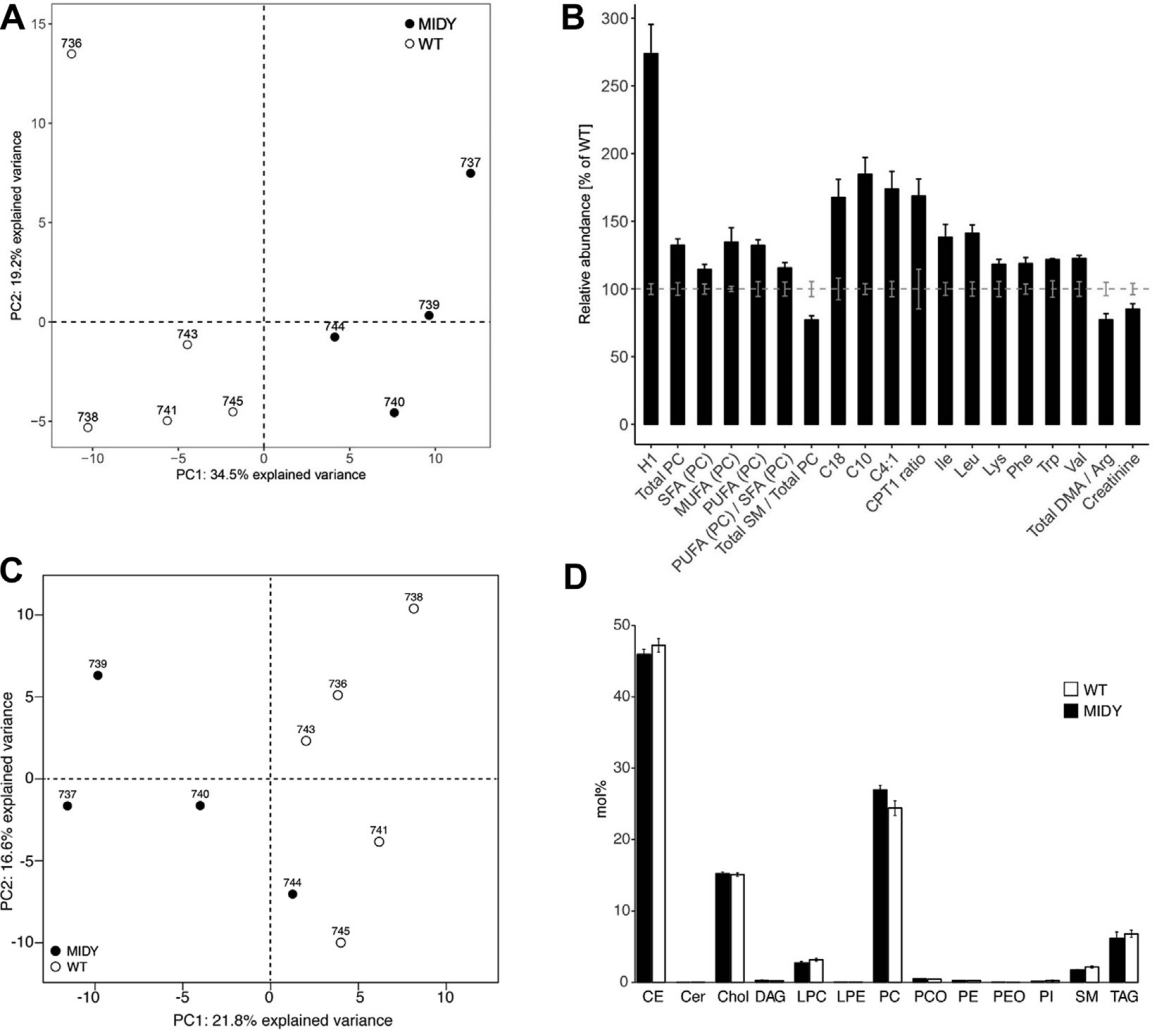
Targeted metabolomics and lipidomics studies of plasma samples from MIDY and WT pigs. **(A**–**B)** Targeted metabolomics. **(A)** Principal component analysis (PCA) is applied to all metabolite concentrations present in Supplementary Table 2 after they were scaled and centered. The bar graph **(B)** shows selected significant (p < 0.05) metabolites and metabolic indicators as a percentage of the WT mean (gray striped line). The SEM for each metabolite and genotype is indicated with error bars. Abbreviations: H1, hexoses; PC, phosphatidylcholine; SFA, saturated fatty acids; MUFA, mono-unsaturated fatty acids; PUFA, poly-unsaturated fatty acids; SM, sphingomyelins; C18, octadecanoylcarnitine; C10, decanoylcarnitine; C4:1, butenylcarnitine; CPT1 ratio, ratio of long chain acylcarnitines to free carnitine; DMA, ratio of dimethylated arginine to total unmodified arginine. **(C**–**D)** Shotgun lipidomics of plasma from MIDY and WT pigs detected 230 lipid species from 13 different classes. **C)** PCA significantly separated MIDY and WT samples (p-value = 0.016). **D)** Mol% abundance of lipid classes in MIDY and WT plasma. Abbreviations: CE, cholesterol esters; Cer, ceramides; Chol, cholesterol; DAG, diacylglycerols; LPC, lysophosphatidylcholines; LPE, lysophosphatidylethanolamine; PC, phosphatidylcholines; PCO, PC plasmalogens; PE, phosphatidylethanolamines; PEO, PE plasmalogens; PI, phosphatidylinositols; SM, sphingomyelins; TAG, triacylglycerols.

Principal component analysis (PCA) of targeted metabolomics data clearly separated the MIDY and WT pig collectives (Figure 2A). Plasma samples of MIDY pigs were characterized by significantly increased levels of hexoses and total phosphatidylcholines (PC), containing a higher proportion of mono- and polyunsaturated than saturated fatty acids. The proportion of sphingomyelins (SM) was significantly reduced. Furthermore, MIDY samples revealed increased concentrations of octadecanoylcarnitine (C18), decanoylcarnitine (C10), and butenylcarnitine (C4:1). The ratio of long chain acylcarnitines to free carnitine (CPT1 ratio) was significantly increased. In addition, the concentrations of branched chain amino acids (valine, leucine, isoleucine), and of lysine, phenylalanine, and tryptophan were significantly increased. The ratio of total dimethylated arginine to total unmodified arginine was significantly decreased in plasma samples from MIDY pigs (Figure 2B). The full set of targeted metabolomics data is shown in Supplementary Table 2.

Since the metabolomics data indicated plasma lipid changes in MIDY pigs, we decided to perform a detailed lipid analysis using shotgun mass spectrometry. Overall, the measurement allowed identifying 230 unique lipid species belonging to 13 lipid classes characteristic for the plasma (the full data can be viewed in Supplementary Table 3). PCA evidently separated MIDY from WT samples (Figure 2C) and confirmed the reduction in plasma SM and an increase of PC in the MIDY samples (Figure 2D). The lipidomics data furthermore showed a decrease in plasma levels of the storage lipids – cholesterol esters (CE) and triacylglycerols (TAG) – in the MIDY pigs.

### 3.2 MIDY pigs exhibit distinct changes in body and organ growth

The body weight of two-year-old MIDY pigs was significantly smaller than that of WT controls (200.3 ± 18.2 kg vs. 238.2 ± 8.7 kg; p = 0.0042). The same was true for body length (172.2 ± 6.8 cm vs. 196.5 ± 12.0 cm; p = 0.0328).

At necropsy, weights and dimensions of a broad spectrum of organs were determined (Supplementary Table 4). MIDY pigs revealed significantly reduced weights of pancreas (97.0 ± 8.3 g vs. 155.2 ± 26.6 g; p = 0.0042), heart (454.5 ± 21.1 g vs. 567.6 ± 45.0 g; p = 0.0025) and *pars proventricularis* of the stomach (265.0 ± 34.2 g vs. 316.0 ± 29.7 g; p = 0.0474). In addition, the lengths of ileum (30.0 ± 4.1 cm vs. 43.0 ± 4.5 cm; p = 0.0028) and cecum (27.5 ± 5.0 cm vs. 36.0 ± 5.5 cm; p = 0.0474) were shorter in MIDY than in WT pigs. Ileum length was also significantly reduced when related to the cube root of body weight (5.2 ± 0.6 cm/kg^1/3^ vs. 7.1 ± 0.8 cm/kg^1/3^; p = 0.0071). Furthermore, there was a trend of reduced relative pancreas weight (0.489 ± 0.073 g/kg vs. 0.654 ± 0.126 g/kg; p = 0.0539). In contrast, relative brain weight was significantly increased in MIDY compared to WT pigs (0.619 ± 0.013 g/kg vs. 0.547 ± 0.046 g/kg; p = 0.0185).

### 3.3 The Munich MIDY Pig Biobank: a comprehensive collection of tissues and body fluids for a broad spectrum of analyses

**Figure 3:**
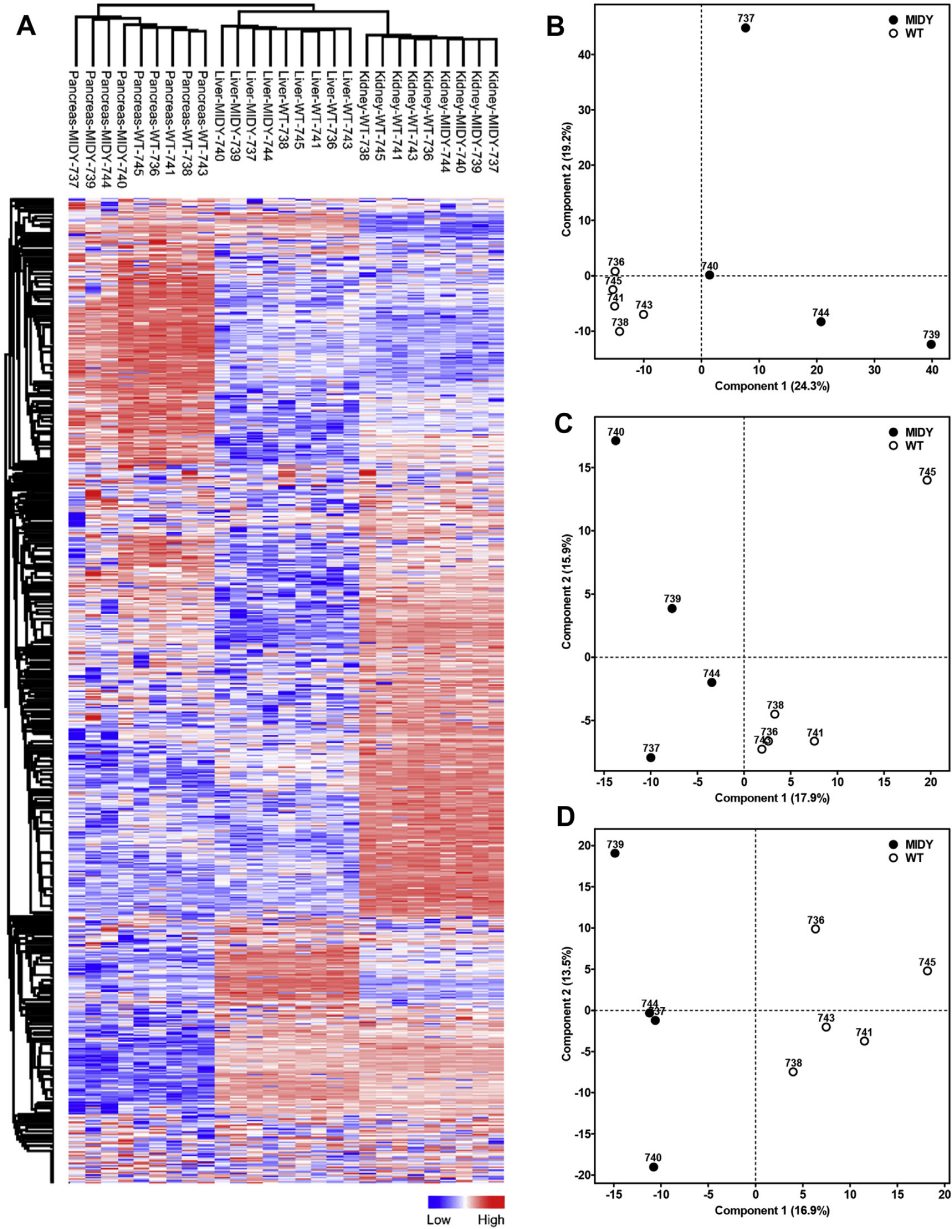
Proteome profiles from a pilot study of pancreas (1574 identified proteins), liver (1263 identified proteins) and kidney cortex (2162 identified proteins) from MIDY and WT pigs. **(A)** Unsupervised hierarchical clustering of normalized expression values (z-score) of 827 proteins commonly identified in pancreas, liver and kidney cortex. The heatmap indicates clustering of the analyzed proteomes according to tissue type and genotype. Missing values were imputed. Heat map legend indicates normalized expression values. **(B**–**D)** Principal component analysis (PCA) of proteomics data from pancreas **(B)**, liver **(C)** and kidney cortex **(D)** clearly separated MIDY and WT samples.

In total, the Munich MIDY Pig Biobank contains approximately 1,900 redundant samples of different body fluids (blood, serum, plasma, urine, cerebrospinal fluid and synovial fluid), as well as ∼17,000 samples from ∼50 different tissues and organs. Table 1 provides an overview of these samples. A detailed list of all individual samples stored in the Munich MIDY Pig Biobank, including the numbers of sampled locations per organ/tissue compartment, as well as the orientation and processing of the specimen is provided in Supplementary Table 5. For a selected set of tissues, RNA was extracted and excellent RNA quality was revealed (Supplementary Figure 2). To demonstrate the suitability of the biobank-tissue samples for protein studies, a pilot proteome analysis of pancreas, liver, and kidney cortex was performed. These analyses showed clear differences between the tissues (Figure 3A) and clustering of MIDY and WT samples within tissue (Figure 3B–D).

## 4 Discussion

Organ crosstalk in diabetes is an area of growing interest [7], [20], [21], [22], but so far, only a limited spectrum of potentially involved organs/tissues was investigated. In view of marked progress in omics technologies for systematic molecular profiling, e.g. on the RNA, protein and metabolite levels, the availability of suitable biological material remains the major limitation for such studies. We thus established a complex biobank from a pig model of MIDY caused by expression of an *INS*^C94Y^ transgene in the beta cells [9]. Wild-type littermates served as controls, providing the best possible genetic control in an outbred large animal model. Since no sex-related differences in the phenotypic consequences of beta cell specific *INS*^C94Y^ expression were noted during the initial characterization of the MIDY pig model [9] and no sex-specific effects were described in human patients with *INS* mutations, we used only female pigs for our long-term study. Fully grown female pigs are much easier to handle compared to adult boars. Expression of mutant insulin C94Y has clear advantages over using streptozotocin (STZ) to induce beta cell death. The individual reaction of pigs towards STZ injection is rather variable leading to a considerable variation in the severity of beta cell damage and diabetic phenotype. Furthermore, GLUT2 through which STZ enters the cell is not only expressed in beta cells but also in liver and kidney tubular cells, resulting in dose-dependent reversible and irreversible damage in these tissues (reviewed in Ref. [8]). In contrast genetically modified MIDY pigs exhibit a stable diabetic phenotype.

Organ-specific, systematic random sampling procedures adapted to porcine biomedical models were applied to determine the tissue sampling locations and sample numbers, ensuring reproducible and representative samples, as a prerequisite for comparative morphologic and multi-omics analyses of a broad spectrum of tissues.

Expression of mutant insulin leads to ER stress and apoptosis of the beta cells. Accordingly, beta cell mass was 70% reduced already in 4.5-month-old MIDY pigs [9] and more than 80% at age two years. In spite of 20% residual beta cell mass, C-peptide was undetectable in the final plasma sample. This indicates that – in addition to the marked reduction of beta cell mass – defective insulin secretion is part of the pathomechanism in MIDY pigs. While insulin treatment restored basal insulin in MIDY pigs to the level of WT, fasting plasma levels of glucose and fructosamine, a valid parameter for the evaluation of medium-term glucose control over 2–3 weeks (reviewed in Ref. [8]), were highly elevated. These findings suggest – in accordance with observations in the Akita mouse model [23] – insulin resistance in MIDY pigs.

Increased plasma glucagon and beta hydroxybutyrate levels as well as a number of characteristic metabolomic changes characterized the 2-year-old MIDY pig with limited insulin treatment as a clinically relevant model of chronic insulin insufficiency and hyperglycemia. Low insulin levels result in activation of hormone-sensitive lipase that releases free fatty acids (FFA) from triglyceride stores. FFA are taken up by the liver and converted to ketone bodies that are released into the circulation (reviewed in Ref. [24]). Carnitine O-palmitoyltransferase 1 (CPT1) transfers the acyl group of long-chain fatty acid-CoA conjugates onto carnitine, a rate-limiting step for mitochondrial uptake of long-chain fatty acids for beta-oxidation. An increased ratio of long-chain acylcarnitines to free carnitine in plasma of MIDY pigs indicates increased activity of CPT1. In addition, the plasma concentrations of several keto- or gluco-/ketogenic amino acids were significantly increased in MIDY pigs. In particular, increased plasma levels of branched-chain amino acids are associated with poor metabolic health and developing or established insulin resistance (reviewed in Ref. [25]).

Reduced body weight and body length of MIDY pigs are explained by their insulin insufficiency, which was not fully compensated by the limited insulin treatment in our study. Without insulin treatment, the body weight of 4.5-month-old MIDY pigs was 40% reduced compared to WT, demonstrating the important anabolic action of insulin. Interestingly, relative brain weight of MIDY pigs was significantly increased, indicating that brain growth is less insulin dependent than overall body growth. This is in line with normal brain development in mice with a neuron-specific inactivation of the insulin receptor (*Insr*) gene [26].

To characterize the quality of samples in the Munich MIDY pig biobank, we extracted and characterized RNA and proteins from a subset of tissues. These pilot studies revealed excellent sample quality. Future detailed omics studies will provide insights into tissue specific molecular changes induced by chronic hyperglycemia and insulin insufficiency. For several tissues, there is already evidence for morphological or functional alterations. A recent study of myocardium showed capillary rarefaction and reduced pericyte investment in 5-month-old MIDY pigs compared to age-matched WT littermates [27]. Additionally, the retina of MIDY pigs (age: 24 or 40 months) exhibited several diabetes associated morphologic alterations [28].

A potential limitation of the Munich MIDY pig biobank is that omics profiles of the samples may be influenced by the drugs used for anesthesia and euthanasia. For instance, ketamine anesthesia is known to activate the sympathetic nervous system and to increase plasma catecholamine concentrations (reviewed in Ref. [29]). Azaperone is at high doses a dopamine receptor blocker [30]. Embutramide as component of T61^®^ is a strong opioid agonist that causes cardiorespiratory depression and can activate opioid receptors in multiple tissues throughout the body, including the central nervous system and the gastrointestinal tract [31]. However, we assume that such effects would affect diabetic and control animals in a similar manner and thus not systematically confound the identification of diabetes-related changes.

In conclusion, the Munich MIDY Pig Biobank facilitates systematic studies of organ crosstalk in diabetes in a multi-organ, multi-omics dimension.

## Funding

This study was supported by the Federal Ministry of Education and Research (Leading-Edge Cluster m^4^ – Personalized Medicine and Targeted Therapies) and by the German Center for Diabetes Research (DZD). M.B and E.L. are supported by DFG fellowships through the Graduate School of Quantitative Biosciences Munich (QBM).

## Author contributions

A.Bl., R.W. and E.W. conceived the experiments. A.Bl. and E.W. wrote the manuscript. S.R., C.P., C.D., A.J., J.A., Ü.C., M.H.d.A., C.S., M.Ri., A.M.L., H.B., G.J.A., T.F. and R.W. contributed to discussions and edited and reviewed the manuscript. S.R., C.B.R. and E.S. performed the metabolic characterization of the MIDY pigs. B.Ra. did the clinical chemical analyses. C.P. and J.A. performed the targeted metabolomics, A.P., M.G., and Ü.C. the lipidomics analyses. F.F., E.L., T.F. and G.J.A. did the pilot proteome studies. S.K. and H.B. analyzed RNA quality. S.H. performed the insulin immunohistochemistry and quantified beta cell mass. E.K. did the multicolor immunofluorescence analysis of islet cell composition. A.Bl., S.R., S.H., C.B.R., B.A., E.S., S.B., A.Bä., A.Br., C.D., E.D.M., M.D., C.E., D.E., R.F., F.G., S.G., A.He., A.Hi., E.K., B.K., M.K., M.L.R., K.M., H.Ö., C.O., M.Re., H.D.R., A.R., B.Ri., M.Ro., M.N.S., A.S., M.R.S., K.S., J.S., N.Ü. and P.Z. performed necropsies, i.e. were assigned to different organ teams and dissected and sampled the respective organs. A.Bl., S.R., F.F., M.B., T.F. and E.W. analyzed the data. A.Bl. and E.W. are the guarantors of this work and, as such, had full access to all the data in the study and take responsibility for the integrity of the data and the accuracy of the data analysis.

## Appendix A Supplementary data

Supplementary Figure 1 Feeding regimen **(a)**, insulin treatment **(b)**, and fasting blood glucose levels **(c)**. Dots in panel c represent the mean fasting blood glucose level of the preceding week. Supplementary Figure 1

Supplementary Figure 2 Quality assessment of RNA derived from various tissues of WT and MIDY pigs. RNA was extracted from heart, skeletal muscle, liver and kidney cortex of five WT and four MIDY pigs. As representative examples, the Agilent Bioanalyzer 2100 profiles obtained for two WT (736 and 738) and two MIDY (737 and 739) pigs are shown. For each sample the calculated RIN and the ratio of absorption at 260 nm and 280 nm derived from UV/VIS spectrometry are indicated. Supplementary Figure 2
