# Supplementary material for: The Munich MIDY Pig Biobank – A unique resource for studying organ crosstalk in diabetes
Source: Mol Metab. 2017 Jun 13;6(8):931–40. doi: 10.1016/j.molmet.2017.06.004 (PMC5518720; doi:10.1016/j.molmet.2017.06.004)
Supplement: Supplementary file 1 [file mmc1.docx]

**Supplementary Table 1.**

**Clinical-chemical and hematological findings in 2-year-old MIDY pigs (n=4) and WT littermate controls (n=5)**

| **Parameter** | **Unit** | **WT** | | **MIDY** | | **P** |
| --- | --- | --- | --- | --- | --- | --- |
|  |  | mean | SD | mean | SD |  |
| Glucose | mg/dl | 120.46 | 25.74 | 310.15 | 39.01 | 0.0001 |
| Fructosamine | µmol/l | 388.60 | 40.24 | 680.00 | 77.53 | 0.0001 |
| Bicarbonate | mmol/l | 26.36 | 4.13 | 26.17 | 1.16 | 0.9196 |
| Beta hydroxybutyrate | µmol/l | 11.00 | 5.83 | 48.25 | 20.36 | 0.0001 |
| Cortisol | nmol/l | 255.20 | 74.58 | 323.50 | 115.17 | 0.0772 |
| Total bilirubin | µmol/l | 0.86 | 0.21 | 1.73 | 0.44 | 0.0224 |
| Urea | mmol/l | 3.06 | 0.65 | 3.00 | 0.14+ | 0.8508 |
| Creatinine | µmol/l | 177 | 14 | 148 | 14 | 0.0151 |
| Total protein | g/l | 66.86 | 3.31 | 68.88 | 1.99 | 0.2977 |
| Albumin | g/l | 43.64 | 3.85 | 43.75 | 1.31 | 0.9547 |
| Sodium | mmol/l | 142.00 | 2.83 | 138.50 | 3.00 | 0.1219 |
| Potassium | mmol/l | 3.84 | 0.24 | 3.81 | 0.08 | 0.7844 |
| Chloride | mmol/l | 100.70 | 1.49 | 97.33 | 1.41 | 0.0109 |
| Calcium | mmol/l | 2.56 | 0.07 | 2.54 | 0.08 | 0.6733 |
| Inorganic phosphorus | mmol/l | 1.88 | 0.16 | 1.83 | 0.05 | 0.5109 |
| Iron | mmol/l | 20.31 | 1.64 | 20.48 | 1.36 | 0.8730 |
| Magnesium | mmol/l | 0.984 | 0.021 | 0.998 | 0.039 | 0.5600 |
| Aspartate aminotransferase | U/l | 87.48 | 45.40 | 44.43 | 16.08 | 0.1037 |
| Gamma glutamyl transferase | U/l | 25.66 | 10.20 | 23.20 | 4.09 | 0.6417 |
| Creatine kinase | U/l | 2946 | 1187 | 3267 | 2810 | 0.8418 |
| Alkaline phosphatase | U/l | 36.00 | 7.62 | 106.00 | 27.78 | 0.0124 |
| Erythrocytes | T/l | 4.55 | 0.65 | 4.78 | 0.25 | 0.4976 |
| Hemoglobin | g/l | 100.40 | 10.95 | 108.25 | 2.06 | 0.1859 |
| Hematocrit | l/l | 0.274 | 0.027 | 0.294 | 0.013 | 0.1899 |
| MCHC^1^ | g/l | 366.20 | 12.40 | 366.80 | 10.76 | 0.9369 |
| Leukocytes | G/l | 13.06 | 2.56 | 9.93 | 1.89 | 0.0621 |
| Thrombocytes | G/l | 155.80 | 40.42 | 140.40 | 72.24 | 0.6913 |

^1^Mean corpuscular hemoglobin concentration.
