# Supplementary material for: The Munich MIDY Pig Biobank – A unique resource for studying organ crosstalk in diabetes
Source: Mol Metab. 2017 Jun 13;6(8):931–40. doi: 10.1016/j.molmet.2017.06.004 (PMC5518720; doi:10.1016/j.molmet.2017.06.004)
Supplement: Supplementary file 2 [file mmc2.docx]

**Supplementary Table 2.**

**Targeted metabolomics findings in 2-year-old MIDY pigs (n=4) and WT littermate controls (n=5)**

| Metabolite | Base [µM] | MIDY [µM] | | WT [µM] | | log2FC | p-value | LOD [µM] | Mean |
| --- | --- | --- | --- | --- | --- | --- | --- | --- | --- |
|  | Mean | Mean | SD | Mean | SD |  |  |  | above LOD |
| C0 | 7.2374 | 6.6577 | 1.3909 | 7.7013 | 1.3767 | -0.1852 | 0.3008 | 2.3709 | TRUE |
| C2 | 1.0577 | 1.0732 | 0.5633 | 1.0453 | 0.3379 | 0.0338 | 0.9342 | 0.1189 | TRUE |
| C3 | 0.1087 | 0.1072 | 0.0280 | 0.1098 | 0.0105 | -0.0305 | 0.8701 | 0.0716 | TRUE |
| C3-OH | 0.0233 | 0.0224 | 0.0033 | 0.0240 | 0.0021 | -0.0871 | 0.4404 | 0.0513 | FALSE |
| C3:1 | 0.0150 | 0.0144 | 0.0027 | 0.0154 | 0.0029 | -0.0829 | 0.6249 | 0.0360 | FALSE |
| C3-DC (C4-OH) | 0.0760 | 0.0742 | 0.0110 | 0.0773 | 0.0055 | -0.0525 | 0.6319 | 0.0362 | TRUE |
| C4 | 0.0678 | 0.0697 | 0.0031 | 0.0662 | 0.0075 | 0.0675 | 0.3733 | 0.0476 | TRUE |
| C4:1 | 0.0478 | 0.0626 | 0.0093 | 0.0360 | 0.0046 | 0.7286 | 0.0058 | 0.0367 | TRUE |
| C5 | 0.0341 | 0.0336 | 0.0085 | 0.0345 | 0.0034 | -0.0344 | 0.8499 | 0.0627 | FALSE |
| C5-OH (C3-DC-M) | 0.0441 | 0.0474 | 0.0100 | 0.0415 | 0.0037 | 0.1718 | 0.3323 | 0.0697 | FALSE |
| C5-M-DC | 0.0282 | 0.0280 | 0.0033 | 0.0284 | 0.0056 | -0.0204 | 0.8854 | 0.0552 | FALSE |
| C5-DC (C6-OH) | 0.0165 | 0.0150 | 0.0023 | 0.0177 | 0.0031 | -0.2091 | 0.1794 | 0.0351 | FALSE |
| C5:1 | 0.0289 | 0.0294 | 0.0010 | 0.0285 | 0.0037 | 0.0403 | 0.6264 | 0.0627 | FALSE |
| C5:1-DC | 0.0122 | 0.0134 | 0.0019 | 0.0113 | 0.0022 | 0.2195 | 0.1703 | 0.0233 | FALSE |
| C6 (C4:1-DC) | 0.0451 | 0.0436 | 0.0055 | 0.0464 | 0.0058 | -0.0812 | 0.4755 | 0.1179 | FALSE |
| C6:1 | 0.0269 | 0.0258 | 0.0026 | 0.0277 | 0.0024 | -0.0894 | 0.3077 | 0.0599 | FALSE |
| C7-DC | 0.0156 | 0.0157 | 0.0034 | 0.0155 | 0.0043 | 0.0157 | 0.9425 | 0.0427 | FALSE |
| C8 | 0.0667 | 0.0681 | 0.0110 | 0.0656 | 0.0059 | 0.0479 | 0.7030 | 0.1321 | FALSE |
| C9 | 0.0213 | 0.0212 | 0.0013 | 0.0213 | 0.0029 | -0.0029 | 0.9742 | 0.0355 | FALSE |
| C10 | 0.1286 | 0.1725 | 0.0233 | 0.0934 | 0.0084 | 0.8103 | 0.0041 | 0.0950 | TRUE |
| C10:1 | 0.0286 | 0.0283 | 0.0042 | 0.0289 | 0.0029 | -0.0276 | 0.8113 | 0.0869 | FALSE |
| C10:2 | 0.0414 | 0.0428 | 0.0030 | 0.0402 | 0.0035 | 0.0783 | 0.2849 | 0.0508 | FALSE |
| C12 | 0.0399 | 0.0432 | 0.0074 | 0.0372 | 0.0051 | 0.1931 | 0.2251 | 0.0928 | FALSE |
| C12-DC | 0.0958 | 0.0950 | 0.0062 | 0.0965 | 0.0033 | -0.0200 | 0.6833 | 0.3288 | FALSE |
| C12:1 | 0.0347 | 0.0360 | 0.0064 | 0.0337 | 0.0041 | 0.0865 | 0.5555 | 0.0861 | FALSE |
| C14 | 0.0112 | 0.0134 | 0.0027 | 0.0094 | 0.0006 | 0.4619 | 0.0563 | 0.0252 | FALSE |
| C14:1 | 0.0148 | 0.0167 | 0.0031 | 0.0133 | 0.0014 | 0.2926 | 0.1107 | 0.0100 | TRUE |
| C14:1-OH | 0.0057 | 0.0060 | 0.0011 | 0.0054 | 0.0005 | 0.1336 | 0.3714 | 0.0165 | FALSE |
| C14:2 | 0.0046 | 0.0050 | 0.0016 | 0.0043 | 0.0006 | 0.2038 | 0.4329 | 0.0111 | FALSE |
| C14:2-OH | 0.0067 | 0.0074 | 0.0011 | 0.0061 | 0.0014 | 0.2435 | 0.1715 | 0.0151 | FALSE |
| C16 | 0.0199 | 0.0239 | 0.0083 | 0.0167 | 0.0032 | 0.4640 | 0.1860 | 0.0140 | TRUE |
| C16-OH | 0.0408 | 0.0442 | 0.0077 | 0.0381 | 0.0110 | 0.1897 | 0.3672 | 0.0104 | TRUE |
| C16:1 | 0.0303 | 0.0312 | 0.0060 | 0.0295 | 0.0017 | 0.0721 | 0.6149 | 0.0838 | FALSE |
| C16:1-OH | 0.0049 | 0.0049 | 0.0017 | 0.0049 | 0.0017 | -0.0043 | 0.9890 | 0.0116 | FALSE |
| C16:2 | 0.0048 | 0.0055 | 0.0004 | 0.0042 | 0.0010 | 0.3306 | 0.0533 | 0.0139 | FALSE |
| C16:2-OH | 0.0120 | 0.0119 | 0.0017 | 0.0121 | 0.0009 | -0.0233 | 0.8257 | 0.0322 | FALSE |
| C18 | 0.0129 | 0.0166 | 0.0027 | 0.0099 | 0.0018 | 0.6787 | 0.0074 | 0.0111 | TRUE |
| C18:1 | 0.0186 | 0.0235 | 0.0114 | 0.0146 | 0.0021 | 0.6309 | 0.2123 | 0.0172 | TRUE |
| C18:1-OH | 0.0099 | 0.0102 | 0.0011 | 0.0097 | 0.0011 | 0.0701 | 0.4933 | 0.0217 | FALSE |
| C18:2 | 0.0065 | 0.0079 | 0.0023 | 0.0053 | 0.0009 | 0.5034 | 0.1103 | 0.0087 | FALSE |
| Ala | 338.2713 | 325.6455 | 62.1903 | 348.3720 | 75.4207 | -0.0862 | 0.6355 | 5.9335 | TRUE |
| Arg | 209.7928 | 208.7214 | 21.5267 | 210.6499 | 30.2321 | -0.0118 | 0.9143 | 0.5000 | TRUE |
| Asn | 28.4667 | 29.4186 | 2.1498 | 27.7052 | 5.0061 | 0.0772 | 0.5175 | 1.5000 | TRUE |
| Asp | 21.7613 | 19.8956 | 4.5666 | 23.2538 | 4.6618 | -0.1982 | 0.3153 | 1.5000 | TRUE |
| Cit | 94.9506 | 91.0230 | 12.9924 | 98.0928 | 17.3954 | -0.0955 | 0.5080 | 1.0000 | TRUE |
| Gln | 425.4086 | 399.1926 | 17.0637 | 446.3814 | 55.7627 | -0.1424 | 0.1346 | 1.5000 | TRUE |
| Glu | 138.0218 | 132.2972 | 16.8762 | 142.6014 | 39.5793 | -0.0958 | 0.6193 | 2.0000 | TRUE |
| Gly | 780.6105 | 634.9126 | 80.3002 | 897.1689 | 216.8401 | -0.4341 | 0.0520 | 0.5000 | TRUE |
| His | 89.5684 | 93.7240 | 17.3921 | 86.2438 | 7.7701 | 0.1071 | 0.4696 | 0.5000 | TRUE |
| Ile | 164.5158 | 194.4627 | 26.4035 | 140.5584 | 15.0006 | 0.4232 | 0.0177 | 1.5739 | TRUE |
| Leu | 231.5973 | 276.4739 | 24.0471 | 195.6960 | 22.8333 | 0.4509 | 0.0018 | 1.5000 | TRUE |
| Lys | 143.5422 | 157.0419 | 9.4924 | 132.7425 | 16.7809 | 0.2175 | 0.0315 | 0.5000 | TRUE |
| Met | 43.0860 | 43.4574 | 9.0899 | 42.7889 | 2.5312 | 0.0199 | 0.8946 | 0.1000 | TRUE |
| Orn | 56.0626 | 61.2988 | 8.9805 | 51.8737 | 7.8145 | 0.2160 | 0.1482 | 0.5000 | TRUE |
| Phe | 84.6960 | 92.8511 | 6.9051 | 78.1720 | 6.5234 | 0.2227 | 0.0161 | 0.1000 | TRUE |
| Pro | 250.2283 | 260.7201 | 21.5167 | 241.8348 | 21.7933 | 0.0968 | 0.2368 | 1.0000 | TRUE |
| Ser | 111.0407 | 111.0086 | 11.0934 | 111.0663 | 13.1849 | -0.0007 | 0.9945 | 0.9120 | TRUE |
| Thr | 144.5532 | 156.6130 | 15.7403 | 134.9053 | 33.2429 | 0.1929 | 0.2449 | 0.5000 | TRUE |
| Trp | 69.5601 | 77.2174 | 1.0825 | 63.4342 | 8.6369 | 0.2548 | 0.0227 | 0.5000 | TRUE |
| Tyr | 77.8017 | 81.4021 | 14.9408 | 74.9213 | 18.9734 | 0.1069 | 0.5844 | 0.5000 | TRUE |
| Val | 313.1438 | 348.9504 | 11.5277 | 284.4986 | 34.3243 | 0.2647 | 0.0107 | 0.5000 | TRUE |
| Ac-Orn | 4.5049 | 3.5690 | 1.2210 | 5.2537 | 1.7055 | -0.4840 | 0.1286 | 0.2000 | TRUE |
| ADMA | 1.0121 | 0.9580 | 0.1327 | 1.0554 | 0.1245 | -0.1234 | 0.3018 | 0.0800 | TRUE |
| alpha-AAA | 11.0105 | 10.4663 | 2.9929 | 11.4459 | 4.6553 | -0.1141 | 0.7141 | 0.4000 | TRUE |
| c4-OH-Pro | 0.0000 | 0.0000 | 0.0000 | 0.0000 | 0.0000 | NA | NA | 0.1000 | FALSE |
| Carnosine | 20.0465 | 18.1588 | 3.0833 | 21.5567 | 2.1351 | -0.2178 | 0.1179 | 0.1000 | TRUE |
| Creatinine | 178.5167 | 162.7676 | 15.0838 | 191.1159 | 18.0928 | -0.2040 | 0.0376 | 1.0000 | TRUE |
| DOPA | 0.0000 | 0.0000 | 0.0000 | 0.0000 | 0.0000 | NA | NA | 0.2000 | FALSE |
| Dopamine | 0.0000 | 0.0000 | 0.0000 | 0.0000 | 0.0000 | NA | NA | 0.1000 | FALSE |
| Histamine | 3.8976 | 4.3084 | 0.4660 | 3.5689 | 1.1273 | 0.2439 | 0.2351 | 0.0100 | TRUE |
| Kynurenine | 0.5910 | 0.5452 | 0.0819 | 0.6277 | 0.1817 | -0.1793 | 0.4004 | 0.1728 | TRUE |
| Met-SO | 1.2685 | 1.2791 | 0.1998 | 1.2600 | 0.2216 | 0.0193 | 0.8958 | 0.3000 | TRUE |
| Nitro-Tyr | 0.0000 | 0.0000 | 0.0000 | 0.0000 | 0.0000 | NA | NA | 0.3000 | FALSE |
| PEA | 0.0000 | 0.0000 | 0.0000 | 0.0000 | 0.0000 | NA | NA | 0.0200 | FALSE |
| Putrescine | 0.3259 | 0.3150 | 0.0397 | 0.3346 | 0.0829 | -0.0773 | 0.6570 | 0.0015 | TRUE |
| SDMA | 0.4078 | 0.3366 | 0.0555 | 0.4647 | 0.1203 | -0.4055 | 0.0798 | 0.3000 | TRUE |
| Serotonin | 3.5082 | 2.8090 | 0.5600 | 4.0676 | 1.3090 | -0.4640 | 0.1035 | 0.0300 | TRUE |
| Spermidine | 0.1359 | 0.1294 | 0.0066 | 0.1411 | 0.0260 | -0.1111 | 0.3787 | 0.1611 | FALSE |
| Spermine | 0.1771 | 0.1668 | 0.0022 | 0.1854 | 0.0282 | -0.1347 | 0.2154 | 0.5000 | FALSE |
| t4-OH-Pro | 26.6909 | 23.4815 | 4.1785 | 29.2584 | 6.3716 | -0.2784 | 0.1472 | 0.1000 | TRUE |
| Taurine | 99.1102 | 90.7420 | 9.9493 | 105.8048 | 13.5586 | -0.1952 | 0.0964 | 0.8000 | TRUE |
| total DMA | 1.0288 | 0.8804 | 0.1079 | 1.1476 | 0.1489 | -0.3346 | 0.0170 | 0.1000 | TRUE |
| lysoPC a C14:0 | 2.6601 | 2.7803 | 0.0652 | 2.5639 | 0.1291 | 0.1044 | 0.0166 | 5.7537 | FALSE |
| lysoPC a C16:0 | 30.6878 | 31.0509 | 6.3045 | 30.3973 | 2.8089 | 0.0273 | 0.8568 | 0.0699 | TRUE |
| lysoPC a C16:1 | 0.9911 | 1.1032 | 0.0715 | 0.9014 | 0.1071 | 0.2619 | 0.0122 | 0.1276 | TRUE |
| lysoPC a C17:0 | 1.3278 | 1.2225 | 0.3436 | 1.4121 | 0.1772 | -0.1834 | 0.3695 | 0.0336 | TRUE |
| lysoPC a C18:0 | 19.7849 | 20.3696 | 5.6029 | 19.3171 | 2.5932 | 0.0682 | 0.7459 | 0.1303 | TRUE |
| lysoPC a C18:1 | 11.4763 | 13.0533 | 1.2254 | 10.2147 | 1.2178 | 0.3185 | 0.0117 | 0.0659 | TRUE |
| lysoPC a C18:2 | 16.9830 | 19.4206 | 1.2006 | 15.0328 | 3.9218 | 0.3328 | 0.0652 | 0.1083 | TRUE |
| lysoPC a C20:3 | 1.3079 | 1.1283 | 0.1615 | 1.4515 | 0.3859 | -0.3181 | 0.1445 | 0.1534 | TRUE |
| lysoPC a C20:4 | 7.2695 | 8.2415 | 1.3102 | 6.4920 | 1.3408 | 0.3098 | 0.0917 | 0.0269 | TRUE |
| lysoPC a C24:0 | 0.7932 | 0.8620 | 0.0622 | 0.7381 | 0.0762 | 0.2006 | 0.0314 | 0.8528 | FALSE |
| lysoPC a C26:0 | 0.6934 | 0.7805 | 0.1609 | 0.6237 | 0.1173 | 0.2911 | 0.1593 | 0.0839 | TRUE |
| lysoPC a C26:1 | 0.3582 | 0.3834 | 0.0649 | 0.3379 | 0.0834 | 0.1630 | 0.3885 | 0.0602 | TRUE |
| lysoPC a C28:0 | 1.0480 | 1.1128 | 0.1326 | 0.9961 | 0.2316 | 0.1430 | 0.3766 | 0.1637 | TRUE |
| lysoPC a C28:1 | 1.2362 | 1.4664 | 0.1492 | 1.0520 | 0.2217 | 0.4331 | 0.0128 | 0.0556 | TRUE |
| PC aa C24:0 | 0.8052 | 0.9265 | 0.0661 | 0.7082 | 0.1289 | 0.3493 | 0.0160 | 0.0526 | TRUE |
| PC aa C26:0 | 2.0759 | 2.3110 | 0.2422 | 1.8878 | 0.4348 | 0.2622 | 0.1110 | 1.2045 | TRUE |
| PC aa C28:1 | 0.6878 | 0.7794 | 0.0818 | 0.6145 | 0.0761 | 0.3086 | 0.0198 | 0.1739 | TRUE |
| PC aa C30:0 | 1.1361 | 1.1832 | 0.1096 | 1.0984 | 0.0683 | 0.0957 | 0.2368 | 0.1354 | TRUE |
| PC aa C30:2 | 0.3118 | 0.3489 | 0.0344 | 0.2821 | 0.0668 | 0.2754 | 0.0992 | 0.0042 | TRUE |
| PC aa C32:0 | 3.2980 | 3.4038 | 0.4962 | 3.2133 | 0.4274 | 0.0741 | 0.5653 | 0.0282 | TRUE |
| PC aa C32:1 | 1.6652 | 1.9420 | 0.5169 | 1.4438 | 0.1023 | 0.3859 | 0.1485 | 0.0090 | TRUE |
| PC aa C32:2 | 0.6265 | 0.7405 | 0.0958 | 0.5353 | 0.0553 | 0.4231 | 0.0148 | 0.0090 | TRUE |
| PC aa C32:3 | 0.1258 | 0.1428 | 0.0202 | 0.1121 | 0.0048 | 0.3149 | 0.0523 | 0.0070 | TRUE |
| PC aa C34:1 | 60.1449 | 70.2812 | 12.0000 | 52.0358 | 2.1408 | 0.3914 | 0.0540 | 0.0705 | TRUE |
| PC aa C34:2 | 91.2992 | 105.5119 | 6.3532 | 79.9290 | 12.7893 | 0.3612 | 0.0077 | 0.0943 | TRUE |
| PC aa C34:3 | 4.4201 | 5.4335 | 0.2825 | 3.6093 | 0.8411 | 0.5353 | 0.0060 | 0.0128 | TRUE |
| PC aa C34:4 | 0.2512 | 0.3074 | 0.0137 | 0.2062 | 0.0347 | 0.5226 | 0.0014 | 0.0113 | TRUE |
| PC aa C36:0 | 0.3253 | 0.3340 | 0.0403 | 0.3182 | 0.1287 | 0.0623 | 0.8061 | 0.1130 | TRUE |
| PC aa C36:1 | 34.9618 | 41.4207 | 5.8497 | 29.7946 | 2.3243 | 0.4296 | 0.0224 | 0.0295 | TRUE |
| PC aa C36:2 | 109.9039 | 129.4485 | 9.3109 | 94.2682 | 19.1980 | 0.4133 | 0.0113 | 0.0566 | TRUE |
| PC aa C36:3 | 24.3847 | 27.9520 | 2.1415 | 21.5308 | 3.7280 | 0.3392 | 0.0158 | 0.0129 | TRUE |
| PC aa C36:4 | 48.4745 | 56.2459 | 6.6844 | 42.2574 | 2.0302 | 0.3721 | 0.0210 | 0.0250 | TRUE |
| PC aa C36:5 | 3.8015 | 4.6895 | 0.4416 | 3.0911 | 0.6498 | 0.5456 | 0.0034 | 0.0106 | TRUE |
| PC aa C36:6 | 0.1105 | 0.1380 | 0.0063 | 0.0885 | 0.0170 | 0.5823 | 0.0015 | 0.0061 | TRUE |
| PC aa C38:0 | 0.4510 | 0.5047 | 0.0285 | 0.4079 | 0.0794 | 0.2762 | 0.0505 | 0.0493 | TRUE |
| PC aa C38:1 | 0.5964 | 0.6732 | 0.0468 | 0.5350 | 0.1228 | 0.2983 | 0.0650 | 0.0086 | TRUE |
| PC aa C38:3 | 22.4562 | 21.7686 | 1.1409 | 23.0063 | 4.9251 | -0.0707 | 0.6121 | 0.0265 | TRUE |
| PC aa C38:4 | 110.9790 | 133.7782 | 19.9984 | 92.7396 | 17.3573 | 0.4786 | 0.0174 | 0.0493 | TRUE |
| PC aa C38:5 | 28.1431 | 33.0113 | 1.5216 | 24.2485 | 3.9921 | 0.4019 | 0.0054 | 0.0216 | TRUE |
| PC aa C38:6 | 6.9348 | 9.0330 | 0.8688 | 5.2563 | 1.2197 | 0.7125 | 0.0010 | 0.0081 | TRUE |
| PC aa C40:1 | 0.2446 | 0.2632 | 0.0302 | 0.2298 | 0.0223 | 0.1751 | 0.1202 | 0.7127 | FALSE |
| PC aa C40:2 | 0.3130 | 0.3604 | 0.0398 | 0.2751 | 0.0290 | 0.3512 | 0.0140 | 0.0347 | TRUE |
| PC aa C40:3 | 0.4583 | 0.4616 | 0.0417 | 0.4556 | 0.0314 | 0.0167 | 0.8211 | 0.0019 | TRUE |
| PC aa C40:4 | 3.8089 | 3.4628 | 0.6861 | 4.0858 | 1.0170 | -0.2101 | 0.3110 | 0.0258 | TRUE |
| PC aa C40:5 | 22.5074 | 23.9301 | 1.9474 | 21.3692 | 2.5021 | 0.1460 | 0.1279 | 0.0400 | TRUE |
| PC aa C40:6 | 5.5281 | 6.5201 | 1.3529 | 4.7344 | 1.2234 | 0.4171 | 0.0843 | 0.2097 | TRUE |
| PC aa C42:0 | 0.1496 | 0.1656 | 0.0122 | 0.1368 | 0.0170 | 0.2476 | 0.0213 | 0.0584 | TRUE |
| PC aa C42:1 | 0.1006 | 0.1117 | 0.0090 | 0.0917 | 0.0095 | 0.2562 | 0.0148 | 0.0177 | TRUE |
| PC aa C42:2 | 0.1193 | 0.1234 | 0.0069 | 0.1161 | 0.0176 | 0.0778 | 0.4356 | 0.0669 | TRUE |
| PC aa C42:4 | 0.0907 | 0.0957 | 0.0015 | 0.0867 | 0.0116 | 0.1284 | 0.1567 | 0.0054 | TRUE |
| PC aa C42:5 | 0.1204 | 0.1298 | 0.0077 | 0.1130 | 0.0223 | 0.1790 | 0.1758 | 0.0114 | TRUE |
| PC aa C42:6 | 0.2941 | 0.3079 | 0.0147 | 0.2830 | 0.0221 | 0.1087 | 0.0839 | 0.4702 | FALSE |
| PC ae C30:0 | 0.2342 | 0.2465 | 0.0195 | 0.2244 | 0.0221 | 0.1207 | 0.1572 | 0.1838 | TRUE |
| PC ae C30:1 | 0.3748 | 0.4129 | 0.0758 | 0.3443 | 0.0873 | 0.2353 | 0.2481 | 0.0073 | TRUE |
| PC ae C30:2 | 0.3021 | 0.3162 | 0.0257 | 0.2909 | 0.0176 | 0.1076 | 0.1516 | 0.5916 | FALSE |
| PC ae C32:1 | 0.9060 | 1.0029 | 0.1083 | 0.8285 | 0.1355 | 0.2475 | 0.0690 | 0.0053 | TRUE |
| PC ae C32:2 | 0.2840 | 0.3041 | 0.0175 | 0.2678 | 0.0280 | 0.1640 | 0.0508 | 0.0223 | TRUE |
| PC ae C34:0 | 0.4229 | 0.4343 | 0.0551 | 0.4138 | 0.0577 | 0.0619 | 0.6062 | 0.0327 | TRUE |
| PC ae C34:1 | 3.1292 | 3.4640 | 0.7082 | 2.8613 | 0.3374 | 0.2476 | 0.1910 | 0.0122 | TRUE |
| PC ae C34:2 | 3.2371 | 3.6972 | 0.5422 | 2.8691 | 0.5781 | 0.3295 | 0.0642 | 0.0062 | TRUE |
| PC ae C34:3 | 1.9622 | 2.3024 | 0.4291 | 1.6900 | 0.4685 | 0.4029 | 0.0816 | 0.0194 | TRUE |
| PC ae C36:0 | 0.2582 | 0.2683 | 0.0468 | 0.2500 | 0.0523 | 0.0912 | 0.5969 | 0.0881 | TRUE |
| PC ae C36:1 | 3.3586 | 3.4558 | 1.0709 | 3.2809 | 0.4557 | 0.0668 | 0.7759 | 0.1183 | TRUE |
| PC ae C36:2 | 6.5416 | 6.8446 | 1.3332 | 6.2993 | 0.8688 | 0.1069 | 0.5116 | 0.0287 | TRUE |
| PC ae C36:3 | 2.1973 | 2.5593 | 0.4186 | 1.9077 | 0.3576 | 0.3825 | 0.0483 | 0.0166 | TRUE |
| PC ae C36:4 | 1.9757 | 2.2783 | 0.2853 | 1.7337 | 0.3095 | 0.3552 | 0.0298 | 0.0098 | TRUE |
| PC ae C36:5 | 1.2788 | 1.5037 | 0.2015 | 1.0990 | 0.1062 | 0.4085 | 0.0193 | 0.0080 | TRUE |
| PC ae C38:0 | 0.5152 | 0.6545 | 0.0525 | 0.4038 | 0.0858 | 0.6340 | 0.0012 | 0.1125 | TRUE |
| PC ae C38:1 | 0.5541 | 0.6039 | 0.1103 | 0.5143 | 0.0734 | 0.2077 | 0.2211 | 0.0051 | TRUE |
| PC ae C38:2 | 1.2441 | 1.2999 | 0.1165 | 1.1994 | 0.2680 | 0.1036 | 0.4808 | 0.0058 | TRUE |
| PC ae C38:3 | 1.3619 | 1.2865 | 0.1301 | 1.4222 | 0.3010 | -0.1279 | 0.4010 | 0.0017 | TRUE |
| PC ae C38:4 | 5.2321 | 5.5341 | 0.8105 | 4.9905 | 0.6341 | 0.1333 | 0.3165 | 0.0092 | TRUE |
| PC ae C38:5 | 2.4331 | 2.8477 | 0.3518 | 2.1015 | 0.3412 | 0.3958 | 0.0167 | 0.0031 | TRUE |
| PC ae C38:6 | 0.5231 | 0.6321 | 0.0771 | 0.4358 | 0.0607 | 0.4859 | 0.0067 | 0.0053 | TRUE |
| PC ae C40:1 | 0.7240 | 0.9485 | 0.1899 | 0.5443 | 0.1511 | 0.7311 | 0.0144 | 0.0122 | TRUE |
| PC ae C40:2 | 0.4301 | 0.4461 | 0.0213 | 0.4173 | 0.0661 | 0.0859 | 0.4015 | 0.0038 | TRUE |
| PC ae C40:3 | 0.4747 | 0.4738 | 0.0379 | 0.4755 | 0.0448 | -0.0047 | 0.9516 | 0.0061 | TRUE |
| PC ae C40:4 | 1.3000 | 1.3404 | 0.0827 | 1.2677 | 0.1472 | 0.0718 | 0.3829 | 0.0796 | TRUE |
| PC ae C40:5 | 1.5325 | 1.5436 | 0.2003 | 1.5236 | 0.2329 | 0.0168 | 0.8937 | 0.0080 | TRUE |
| PC ae C40:6 | 0.5097 | 0.5829 | 0.0607 | 0.4512 | 0.0734 | 0.3328 | 0.0216 | 0.0081 | TRUE |
| PC ae C42:0 | 0.5830 | 0.5960 | 0.0238 | 0.5727 | 0.0212 | 0.0513 | 0.1754 | 1.3755 | FALSE |
| PC ae C42:1 | 0.4786 | 0.5472 | 0.0771 | 0.4237 | 0.0502 | 0.3324 | 0.0398 | 0.1838 | TRUE |
| PC ae C42:2 | 0.5331 | 0.6215 | 0.0567 | 0.4623 | 0.1075 | 0.3853 | 0.0277 | 0.0125 | TRUE |
| PC ae C42:3 | 0.1583 | 0.1878 | 0.0265 | 0.1348 | 0.0241 | 0.4326 | 0.0199 | 0.0016 | TRUE |
| PC ae C42:4 | 0.1385 | 0.1346 | 0.0096 | 0.1417 | 0.0141 | -0.0658 | 0.4012 | 0.3000 | FALSE |
| PC ae C42:5 | 0.7915 | 0.8138 | 0.0411 | 0.7737 | 0.0433 | 0.0650 | 0.2005 | 1.5056 | FALSE |
| PC ae C44:3 | 0.1099 | 0.1170 | 0.0222 | 0.1042 | 0.0200 | 0.1501 | 0.4015 | 0.0249 | TRUE |
| PC ae C44:4 | 0.0881 | 0.0947 | 0.0093 | 0.0828 | 0.0092 | 0.1732 | 0.1008 | 0.1042 | FALSE |
| PC ae C44:5 | 0.1031 | 0.1151 | 0.0123 | 0.0935 | 0.0131 | 0.2693 | 0.0399 | 0.1166 | FALSE |
| PC ae C44:6 | 0.1147 | 0.1213 | 0.0265 | 0.1094 | 0.0111 | 0.1334 | 0.4483 | 0.0510 | TRUE |
| SM (OH) C14:1 | 2.1201 | 2.0191 | 0.4285 | 2.2010 | 0.4080 | -0.1101 | 0.5405 | 0.0250 | TRUE |
| SM (OH) C16:1 | 2.5365 | 2.3309 | 0.1492 | 2.7009 | 0.5583 | -0.1873 | 0.2184 | 0.0120 | TRUE |
| SM (OH) C22:1 | 3.3428 | 3.4258 | 0.4093 | 3.2764 | 0.7549 | 0.0573 | 0.7176 | 0.0312 | TRUE |
| SM (OH) C22:2 | 1.4918 | 1.3815 | 0.2504 | 1.5801 | 0.3092 | -0.1709 | 0.3224 | 0.0064 | TRUE |
| SM (OH) C24:1 | 0.4193 | 0.4216 | 0.0608 | 0.4175 | 0.1018 | 0.0123 | 0.9437 | 0.0186 | TRUE |
| SM C16:0 | 48.1126 | 49.7431 | 5.0082 | 46.8082 | 6.4729 | 0.0782 | 0.4683 | 0.0293 | TRUE |
| SM C16:1 | 5.0172 | 5.2457 | 0.7632 | 4.8345 | 0.9987 | 0.1051 | 0.5065 | 0.0110 | TRUE |
| SM C18:0 | 10.2014 | 10.7454 | 1.5076 | 9.7661 | 2.1746 | 0.1232 | 0.4526 | 0.0700 | TRUE |
| SM C18:1 | 2.7953 | 2.9899 | 0.5475 | 2.6396 | 0.4257 | 0.1609 | 0.3365 | 0.0117 | TRUE |
| SM C20:2 | 0.1603 | 0.1879 | 0.0342 | 0.1382 | 0.0363 | 0.3995 | 0.0747 | 0.0050 | TRUE |
| SM C22:3 | 0.0777 | 0.0967 | 0.0404 | 0.0626 | 0.0643 | 0.5706 | 0.3645 | 0.0088 | TRUE |
| SM C24:0 | 8.8066 | 8.4521 | 0.7790 | 9.0901 | 1.5624 | -0.0929 | 0.4551 | 0.0382 | TRUE |
| SM C24:1 | 10.4813 | 9.5190 | 1.0100 | 11.2511 | 2.5915 | -0.2123 | 0.2248 | 0.0151 | TRUE |
| SM C26:0 | 0.1009 | 0.0943 | 0.0386 | 0.1061 | 0.0325 | -0.1501 | 0.6430 | 0.0150 | TRUE |
| SM C26:1 | 0.0809 | 0.0682 | 0.0585 | 0.0910 | 0.0547 | -0.3625 | 0.5715 | 0.0103 | TRUE |
| H1 | 11239.8160 | 17367.5027 | 2717.7073 | 6337.6666 | 566.2124 | 1.3488 | 0.0032 | 20.0000 | TRUE |
| C2 / C0 | 0.1446 | 0.1545 | 0.0561 | 0.1366 | 0.0433 | 0.1588 | 0.6197 | NA | NA |
| (C2+C3) / C0 | 0.1597 | 0.1706 | 0.0569 | 0.1511 | 0.0432 | 0.1569 | 0.5924 | NA | NA |
| CPT1 ratio | 0.0047 | 0.0061 | 0.0009 | 0.0036 | 0.0012 | 0.6877 | 0.0092 | NA | NA |
| Total AC / C0 | 0.3230 | 0.3594 | 0.0473 | 0.2939 | 0.0533 | 0.2607 | 0.0928 | NA | NA |
| Total AC-DC /  Total AC | 0.0754 | 0.0726 | 0.0163 | 0.0777 | 0.0138 | -0.0871 | 0.6341 | NA | NA |
| Total AC-OH /  Total AC | 0.0803 | 0.0790 | 0.0175 | 0.0814 | 0.0141 | -0.0386 | 0.8301 | NA | NA |
| SFA (PC) | 10.2545 | 11.0284 | 0.7422 | 9.6354 | 0.8095 | 0.1744 | 0.0321 | NA | NA |
| MUFA (PC) | 107.9266 | 125.9065 | 19.8317 | 93.5426 | 4.0000 | 0.3869 | 0.0447 | NA | NA |
| MUFA (PC) /  SFA (PC) | 10.4841 | 11.3841 | 1.2523 | 9.7641 | 0.9407 | 0.1985 | 0.0797 | NA | NA |
| PUFA (PC) | 520.3203 | 601.9406 | 37.6146 | 455.0240 | 55.8785 | 0.3640 | 0.0023 | NA | NA |
| PUFA (PC) /  SFA (PC) | 50.6018 | 54.6959 | 3.7963 | 47.3265 | 5.4520 | 0.1870 | 0.0489 | NA | NA |
| PUFA (PC) /  MUFA (PC) | 4.8532 | 4.8437 | 0.5805 | 4.8608 | 0.5205 | -0.0045 | 0.9647 | NA | NA |
| Total PC | 638.5014 | 738.8756 | 52.1461 | 558.2020 | 58.4719 | 0.3648 | 0.0019 | NA | NA |
| Total lysoPC | 96.6172 | 102.9753 | 14.4537 | 91.5306 | 11.2188 | 0.1520 | 0.2442 | NA | NA |
| Total lysoPC /  Total PC | 0.1531 | 0.1398 | 0.0221 | 0.1637 | 0.0048 | -0.2006 | 0.1175 | NA | NA |
| Total PC aa | 592.1051 | 688.2423 | 47.8197 | 515.1954 | 56.7143 | 0.3769 | 0.0017 | NA | NA |
| Total PC ae | 46.3962 | 50.6332 | 5.8559 | 43.0066 | 3.7999 | 0.2112 | 0.0746 | NA | NA |
| Total SM | 95.7447 | 96.7210 | 9.7149 | 94.9636 | 14.9969 | 0.0235 | 0.8381 | NA | NA |
| Total (PC+SM) | 734.2460 | 835.5966 | 57.6470 | 653.1656 | 69.4553 | 0.3199 | 0.0036 | NA | NA |
| Total SM /  Total (SM+PC) | 0.1321 | 0.1158 | 0.0086 | 0.1451 | 0.0154 | -0.2854 | 0.0100 | NA | NA |
| Total SM /  Total PC | 0.1527 | 0.1310 | 0.0110 | 0.1700 | 0.0213 | -0.3288 | 0.0114 | NA | NA |
| Total SM-non OH | 85.8341 | 87.1422 | 9.2616 | 84.7876 | 13.2570 | 0.0352 | 0.7635 | NA | NA |
| Total SM-OH | 9.9106 | 9.5788 | 0.7428 | 10.1760 | 1.8895 | -0.0773 | 0.5440 | NA | NA |
| Total SM-OH /  Total SM-non OH | 0.1156 | 0.1104 | 0.0100 | 0.1197 | 0.0093 | -0.1032 | 0.2019 | NA | NA |
| Orn / Arg | 0.2698 | 0.2963 | 0.0551 | 0.2487 | 0.0419 | 0.2269 | 0.2068 | NA | NA |
| Tyr / Phe | 0.9147 | 0.8723 | 0.0979 | 0.9487 | 0.1787 | -0.1071 | 0.4446 | NA | NA |
| AAA | 232.0578 | 251.4707 | 21.1669 | 216.5275 | 32.7382 | 0.1934 | 0.0956 | NA | NA |
| BCAA | 709.2570 | 819.8870 | 61.1177 | 620.7529 | 70.9901 | 0.3619 | 0.0028 | NA | NA |
| ESAA | 1284.2629 | 1440.7919 | 104.1715 | 1159.0397 | 115.9403 | 0.2822 | 0.0067 | NA | NA |
| GAA | 1229.9226 | 1071.5667 | 133.8161 | 1356.6073 | 280.2365 | -0.2983 | 0.0919 | NA | NA |
| KAA | 916.2664 | 1036.0622 | 84.4783 | 820.4297 | 92.5453 | 0.3029 | 0.0086 | NA | NA |
| BCAA/AAA | 3.0635 | 3.2656 | 0.1837 | 2.9018 | 0.3981 | 0.1524 | 0.1204 | NA | NA |

Metabolomics data was analyzed in R (version 3.3.2) using RStudio. Means and standard deviations (SD) were calculated, and student t-tests were performed using the respective base R functions. For calculation of the batch specific limit of detection (LOD) for individual metabolites the three PBS based Zero Samples were used. The LOD was determined by taking the median of the metabolite concentration in the Zero Samples multiplied by three. In the case that the metabolite was not detected in the Zero Sample, as is the case for most amino acids and biogenic amines, the kit predetermined LOD was used instead (the measurement method of amino acids and biogenic amines are done by LC-MS/MS, everything else is done by FIA-MS/MS, thus the different methods have varied levels of sensitivity).
