# Supplementary material for: The Munich MIDY Pig Biobank – A unique resource for studying organ crosstalk in diabetes
Source: Mol Metab. 2017 Jun 13;6(8):931–40. doi: 10.1016/j.molmet.2017.06.004 (PMC5518720; doi:10.1016/j.molmet.2017.06.004)
Supplement: Supplementary file 4 [file mmc4.docx]

**Supplementary Table 4.**

**Body and organ weights/dimensions of 2-year-old MIDY pigs (n=4) and WT littermate controls (n=5)**

|  | **Absolute values** | | | | | |  | **Relative values^1^** | | | | | |
| --- | --- | --- | --- | --- | --- | --- | --- | --- | --- | --- | --- | --- | --- |
| **Parameter** | **Unit** | **WT** | | **MIDY** | | **P** |  | **Unit** | **WT** | | **MIDY** | | **P** |
|  |  | mean | SD | mean | SD |  |  |  | mean | SD | mean | SD |  |
| Body weight | kg | 238.2 | 8.7 | 200.25 | 18.2 | 0.0042 |  |  |  |  |  |  |  |
| Body length | cm | 196.5 | 12.0 | 172.2 | 6.8 | 0.0328 |  | cm/kg^1/3^ | 32.171 | 2.062 | 30.252 | 1.875 | 0.2989 |
| Pancreas weight | g | 155.2 | 26.6 | 97.0 | 8.3 | 0.0042 |  | g/kg | 0.654 | 0.126 | 0.489 | 0.073 | 0.0539 |
| Kidney weight^2^ | g | 249.6 | 42.2 | 258.8 | 53.1 | 0.7810 |  | g/kg | 1.049 | 0.177 | 1.291 | 0.216 | 0.1065 |
| Spleen weight | g | 855.0 | 403.0 | 846.3 | 346.9 | 0.9730 |  | g/kg | 3.580 | 1.469 | 4.245 | 1.842 | 0.5861 |
| Liver weight | g | 2808.0 | 786.2 | 2505.0 | 313.0 | 0.4957 |  | g/kg | 11.793 | 3.258 | 12.600 | 2.035 | 0.6804 |
| Lung weight | g | 1068.0 | 138.1 | 874.3 | 253.7 | 0.1844 |  | g/kg | 4.478 | 0.490 | 4.334 | 0.977 | 0.7810 |
| Heart weight | g | 567.6 | 45.0 | 454.5 | 21.1 | 0.0025 |  | g/kg | 2.387 | 0.224 | 2.278 | 0.138 | 0.4273 |
| Brain weight | g | 130.3 | 12.7 | 124.0 | 10.3 | 0.4466 |  | g/kg | 0.547 | 0.046 | 0.619 | 0.013 | 0.0185 |
| Stomach weight | g | 1176.0 | 194.6 | 970.0 | 77.5 | 0.0891 |  | g/kg | 4.958 | 0.977 | 4.873 | 0.568 | 0.8832 |
| *P. proventricul.* | g | 316.0 | 29.7 | 265.0 | 34.2 | 0.0474 |  | g/kg | 1.328 | 0.130 | 1.337 | 0.250 | 0.9446 |
| *P. glandularis* | g | 456.0 | 110.8 | 380.0 | 54.2 | 0.2530 |  | g/kg | 1.927 | 0.542 | 1.901 | 0.227 | 0.9311 |
| *P. pyloris* | g | 404.0 | 82.9 | 325.0 | 41.2 | 0.1282 |  | g/kg | 1.703 | 0.395 | 1.635 | 0.287 | 0.7835 |
| Stomach area | cm^2^ | 1074.7 | 199.7 | 905.8 | 138.4 | 0.1956 |  | cm^2^/kg^2/3^ | 29.107 | 6.095 | 27.354 | 3.079 | 0.6193 |
| *Fundus* | cm^2^ | 523.7 | 138.0 | 423.0 | 83.8 | 0.2388 |  | cm^2^/kg^2/3^ | 14.204 | 4.109 | 12.738 | 2.047 | 0.5388 |
| *Cardia* | cm^2^ | 296.8 | 24.9 | 256.2 | 96.4 | 0.3882 |  | cm^2^/kg^2/3^ | 8.024 | 0.798 | 7.726 | 2.644 | 0.8160 |
| *Pylorus* | cm^2^ | 254.1 | 46.6 | 227.6 | 42.5 | 0.4048 |  | cm^2^/kg^2/3^ | 6.880 | 1.400 | 6.889 | 1.246 | 0.9914 |
| Intestine weight | g | 5510.0 | 597.4 | 4852.5 | 328.8 | 0.0909 |  | g/kg | 23.213 | 3.306 | 24.280 | 0.925 | 0.5558 |
| Duodenum | g | 84.0 | 40.9 | 37.5 | 12.6 | 0.0674 |  | g/kg | 0.355 | 0.174 | 0.191 | 0.075 | 0.1274 |
| Jejunum | g | 2340.0 | 554.8 | 2055.0 | 112,4 | 0.3516 |  | g/kg | 9.893 | 2.743 | 10.288 | 0.395 | 0.7860 |
| Ileum | g | 86.0 | 45.6 | 55.0 | 10.0 | 0.2293 |  | g/kg | 0.361 | 0.189 | 0.278 | 0.066 | 0.4331 |
| Caecum | g | 260.0 | 28.3 | 242.5 | 81.0 | 0.6620 |  | g/kg | 1.092 | 0.117 | 1.228 | 0.474 | 0.5504 |
| Colon | g | 2740.0 | 115.8 | 2462.5 | 243.1 | 0.0568 |  | g/kg | 11.512 | 0.573 | 12.294 | 0.366 | 0.0507 |
| Intestine length | cm | 2360.0 | 220.8 | 2100.0 | 233.2 | 0.1303 |  | cm/kg^1/3^ | 388.1 | 39.7 | 365.2 | 33.6 | 0.3909 |
| Duodenum | cm | 43.0 | 13.0 | 35.0 | 8.2 | 0.3228 |  | cm/kg^1/3^ | 7.1 | 2.2 | 6.1 | 1.6 | 0.4880 |
| Jejunum | cm | 1600.0 | 187.1 | 1432.5 | 102.8 | 0.1549 |  | cm/kg^1/3^ | 263.2 | 33.3 | 249.3 | 13.3 | 0.4622 |
| Ileum | cm | 43.0 | 4.5 | 30.0 | 4.1 | 0.0028 |  | cm/kg^1/3^ | 7.1 | 0.8 | 5.2 | 0.6 | 0.0071 |
| Caecum | cm | 36.0 | 5.5 | 27.5 | 5.0 | 0.0474 |  | cm/kg^1/3^ | 5.9 | 0.9 | 4.8 | 0.8 | 0.0946 |
| Colon | cm | 638.0 | 113.9 | 575.0 | 150.0 | 0.4954 |  | cm/kg^1/3^ | 104.8 | 18.6 | 99.9 | 24.2 | 0.7359 |
| Uterus | g | 1328.8 | 558.9 | 1260.5 | 203.0 | 0.8248 |  | g/kg | 5.604 | 2.407 | 6.285 | 0.681 | 0.6049 |
| Ovaries^3^ | g | 41.8 | 20.7 | 105.5 | 138.8 | 0.3373 |  | g/kg | 0.178 | 0.096 | 0.497 | 0.640 | 0.2991 |
| Thyroid gland | g | 15.5 | 4.2 | 13.9 | 2.6 | 0.5036 |  | g/kg | 0.065 | 0.018 | 0.069 | 0.010 | 0.7105 |

^1^Area and length measurements were related to square root and cube root of body weight respectively to keep the same dimension. ^2^Weight of the right, non-perfused kidney. ^3^Cumulative weight of left and right ovary. Intestinal weight was determined without ingesta/feces.
