# Supplementary figures and images for: The Munich MIDY Pig Biobank – A unique resource for studying organ crosstalk in diabetes
Source: Mol Metab. 2017 Jun 13;6(8):931–40. doi: 10.1016/j.molmet.2017.06.004 (PMC5518720; doi:10.1016/j.molmet.2017.06.004)

## Slide 1
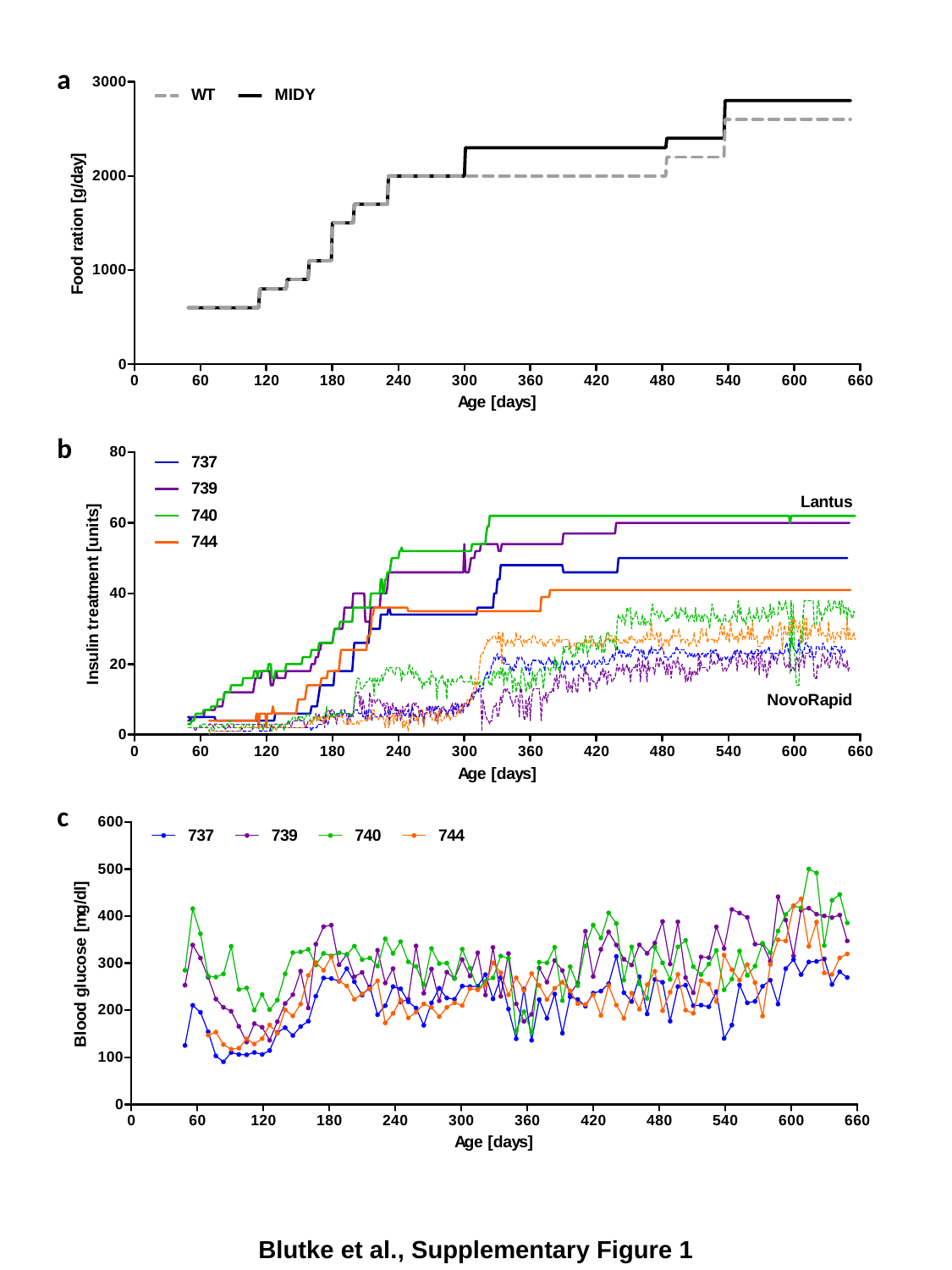

a
b
c
Blutke et al., Supplementary Figure 1

Supplement: Supplementary Figure 1 — Feeding regimen (a), insulin treatment (b), and fasting blood glucose levels (c). Dots in panel c represent the mean fasting blood glucose level of the preceding week. [file mmc6.pptx]
