# Supplementary material for: The Munich MIDY Pig Biobank – A unique resource for studying organ crosstalk in diabetes
Source: Mol Metab. 2017 Jun 13;6(8):931–40. doi: 10.1016/j.molmet.2017.06.004 (PMC5518720; doi:10.1016/j.molmet.2017.06.004)
Supplement: Supplementary Figure 2 — Quality assessment of RNA derived from various tissues of WT and MIDY pigs. RNA was extracted from heart, skeletal muscle, liver and kidney cortex of five WT and four MIDY pigs. As representative examples, the Agilent Bioanalyzer 2100 profiles obtained for two WT (736 and 738) and two MIDY (737 and 739) pigs are shown. For each sample the calculated RIN and the ratio of absorption at 260 nm and 280 nm derived from UV/VIS spectrometry are indicated. [file mmc7.pptx]

## Slide 1
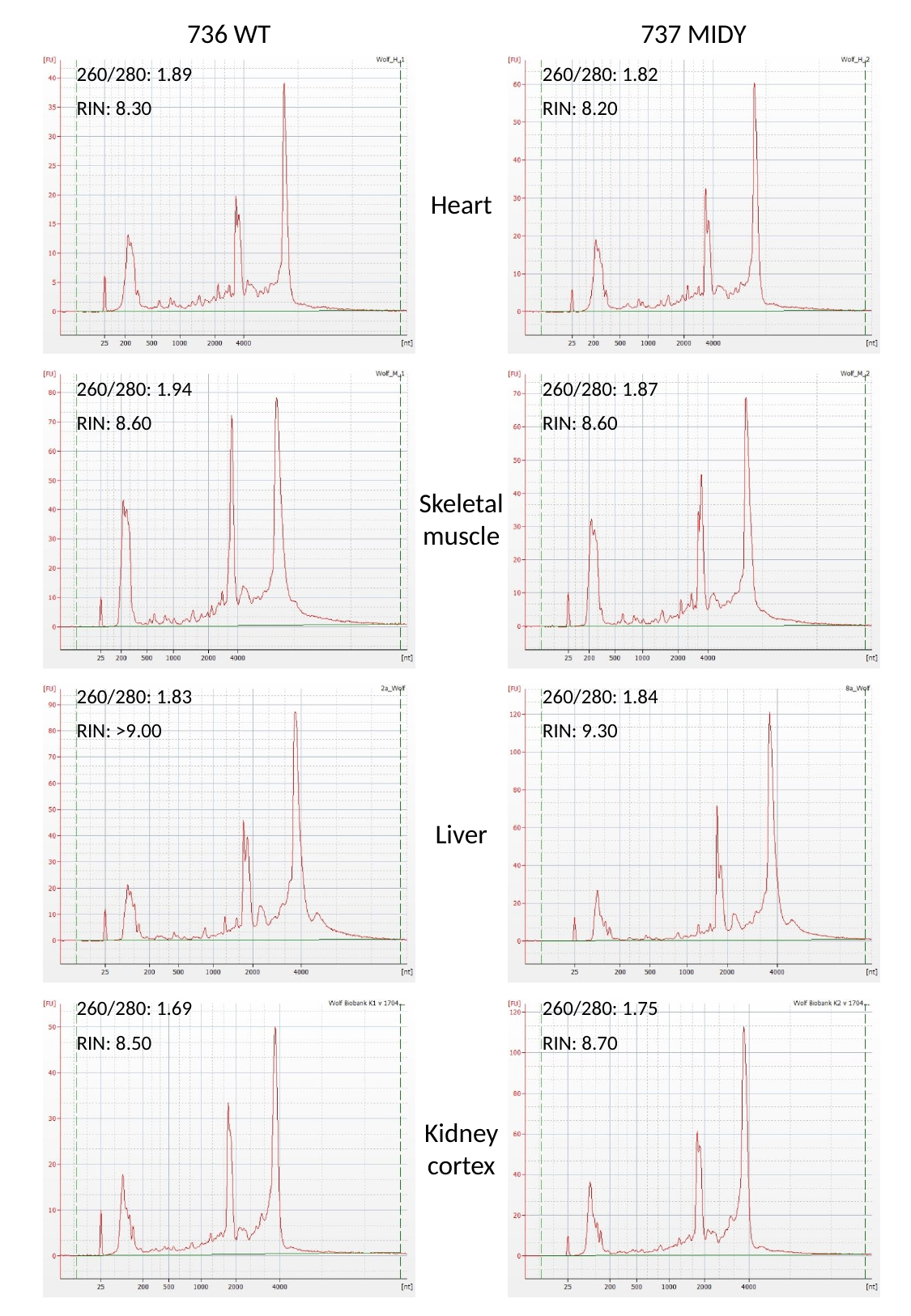

736 WT
737 MIDY
260/280: 1.89
RIN: 8.30
260/280: 1.82
RIN: 8.20
Heart
260/280: 1.94
RIN: 8.60
260/280: 1.87
RIN: 8.60
Skeletal
muscle
260/280: 1.83
RIN: >9.00
260/280: 1.84
RIN: 9.30
Liver
260/280: 1.69
RIN: 8.50
260/280: 1.75
RIN: 8.70
Kidney
cortex

## Slide 2
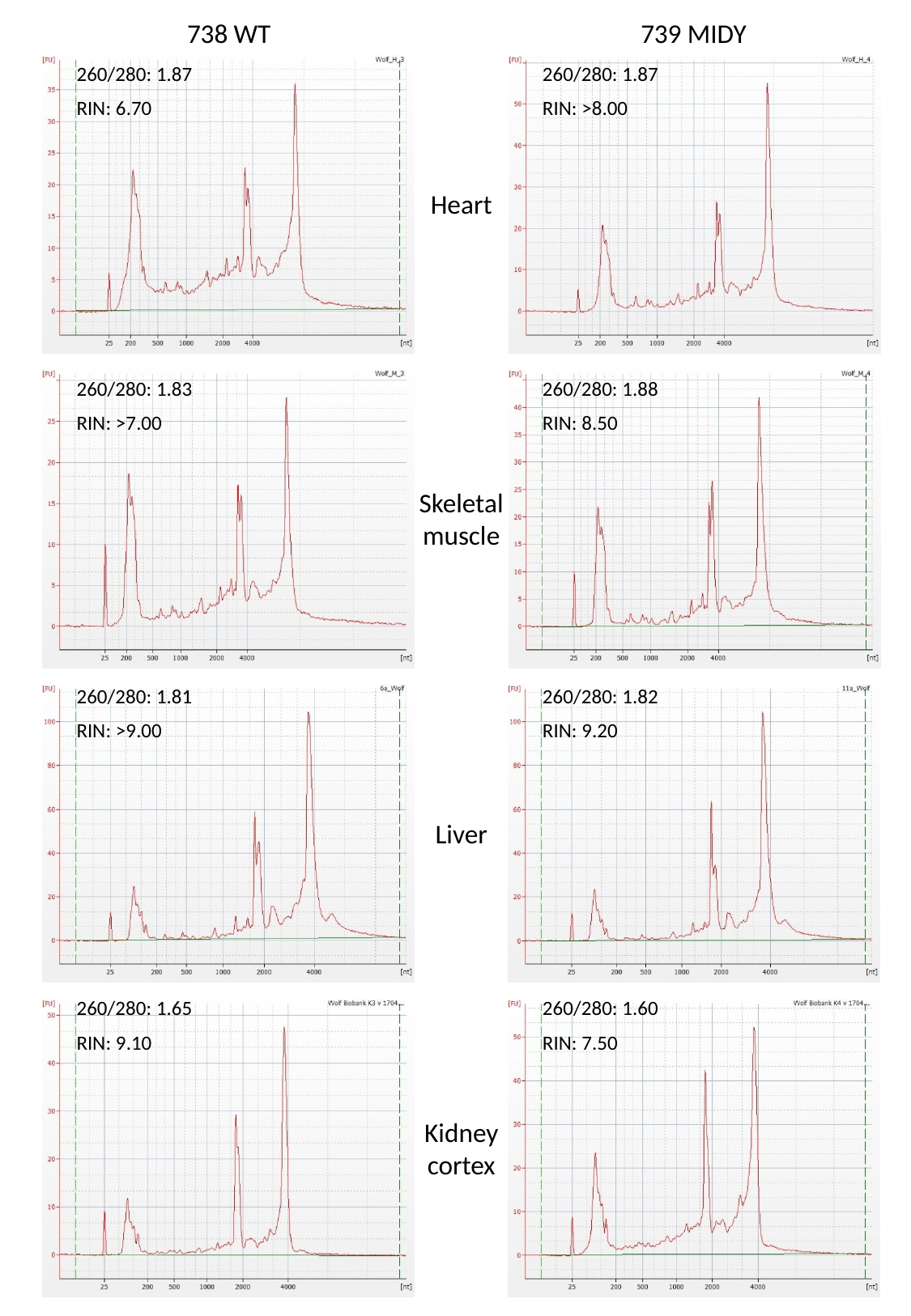

738 WT
739 MIDY
260/280: 1.87
RIN: 6.70
260/280: 1.87
RIN: >8.00
Heart
260/280: 1.83
RIN: >7.00
260/280: 1.88
RIN: 8.50
Skeletal
muscle
260/280: 1.81
RIN: >9.00
260/280: 1.82
RIN: 9.20
Liver
260/280: 1.65
RIN: 9.10
260/280: 1.60
RIN: 7.50
Kidney
cortex
